# *Leishmania infantum* infection does not affect the main composition of the intestinal microbiome of the Syrian hamster

**DOI:** 10.1186/s13071-022-05576-1

**Published:** 2022-12-15

**Authors:** Ana Isabel Olías-Molero, Pedro Botías, Montserrat Cuquerella, Jesús García-Cantalejo, Emilia Barcia, Susana Torrado, Juan José Torrado, José María Alunda

**Affiliations:** 1 ICPVet, Department of Animal Health, School of Veterinary Sciences, Complutense University of Madrid, Madrid, Spain; 2 Unidad de Genómica, Centro de Asistencia a la Investigación de Técnicas Biológicas, Complutense University of Madrid, Madrid, Spain; 3 Department of Pharmaceutics and Food Technology, School of Pharmacy, Complutense University of Madrid, Madrid, Spain; 4 Institute of Industrial Pharmacy UCM, School of Pharmacy, Complutense University of Madrid, Madrid, Spain

**Keywords:** *Leishmania infantum*, Intestinal microbiome, Syrian hamster, Infection, 16S metagenomics

## Abstract

**Background:**

Visceral leishmaniasis (VL) is the most severe form of all leishmanial infections and is caused by infection with protozoa of *Leishmania donovani* and *Leishmania infantum*. This parasitic disease occurs in over 80 countries and its geographic distribution is on the rise. Although the interaction between the intestinal microbiome and the immune response has been established in several pathologies, it has not been widely studied in leishmaniasis. The Syrian hamster is the most advanced laboratory model for developing vaccines and new drugs against VL. In the study reported here, we explored the relationship between the intestinal microbiome and infection with *L. infantum* in this surrogate host.

**Methods:**

Male Syrian hamsters (120–140 g) were inoculated with 10^8^ promastigotes of a canine-derived *L. infantum* strain or left as uninfected control animals. Infection was maintained for 19 weeks (endpoint) and monitored by an immunoglobulin G (IgG) enyzme-linked immunosorbent assay throughout the experiment. Individual faecal samples, obtained at weeks 16, 18 and 19 post-inoculation, were analysed to determine the 16S metagenomic composition (the operational taxonomic units [OTUs] of the intestinal microbiome and the comparison between groups were FDR (false discovery rate)-adjusted).

**Results:**

*Leishmania infantum* infection elicited moderate clinical signs and lesions and a steady increase in specific anti-*Leishmania* serum IgG. The predominant phyla (*Firmicutes* + *Bacteriodetes*: > 90%), families (*Muribaculaceae* + *Lachnospiraceae* + *Ruminococcaceae*: 70–80%) and genera found in the uninfected hamsters showed no significant variations throughout the experiment. *Leishmania infantum* infection provoked a slightly higher—albeit non-significant—value for the *Firmicutes/Bacteriodetes* ratio but no notable differences were found in the relative abundance or diversity of phyla and families. The microbiome of the infected hamsters was enriched in *CAG-352*, whereas *Lachnospiraceae UCG-004*, the *[Eubacterium] ventriosum* group and *Allobaculum* were less abundant.

**Conclusions:**

The lack of extensive significant differences between hamsters infected and uninfected with *L. infantum* in the higher taxa (phyla, families) and the scarce variation found, which was restricted to genera with a low relative abundance, suggest that there is no clear VL infection-intestinal microbiome axis in hamsters. Further studies are needed (chronic infections, co-abundance analyses, intestinal sampling, functional analysis) to confirm these findings and to determine more precisely the possible relationship between microbiome composition and VL infection.

**Graphical Abstract:**

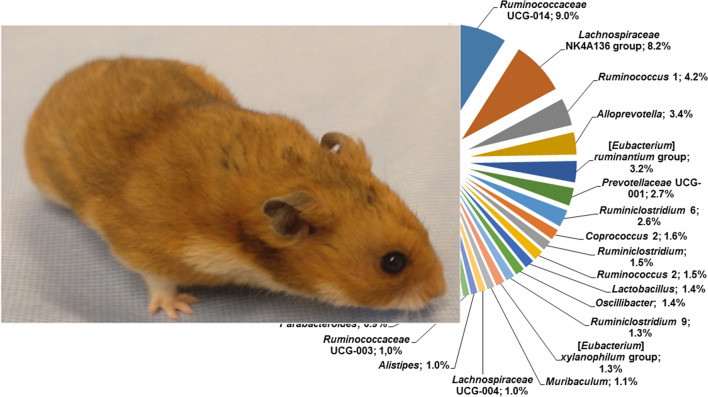

**Supplementary Information:**

The online version contains supplementary material available at 10.1186/s13071-022-05576-1.

## Background

Leishmaniasis refers to a broad group of vector-borne parasitic diseases caused by species of the genus *Leishmania*. Infection can occur in humans and other mammals and causes a range of clinical presentations (visceral, cutaneous, mucocutaneous, post-kala-azar dermal leishmaniasis) [1]. The most severe disease is visceral leishmaniasis (VL), which is fatal unless treated, and is caused by *Leishmania donovani* and *Leishmania infantum* (= *L. chagasi*) [2, 3]. The geographic distribution of VL is increasing due to human migration and travel, as well as the spread of vector populations to previously unaffected regions [4]. The infection is also frequent in HIV-positive patients in endemic areas [5–7] and has been reported in recipients of solid organ transplants [8, 9]. While infections by *L. donovani* are regarded as anthroponotic, leishmaniasis caused by *L. infantum* is zoonotic (zoonotic VL), and dogs are considered to be the main reservoir of human disease [10–12]. Disease outcome after inoculation depends on the inherent virulence of the *Leishmania* strain, individual immune response, host health status and intercurrent infections, among other factors [13, 14]. The Syrian hamster is the most advanced surrogate model for *Leishmania* infections, particularly those caused by *L. donovani* and *L. infantum*, and is widely used to study pathogenicity and experimental chemotherapy [15–20].

There is a growing awareness of the importance of gut microbiota for general health status [21]. It has been shown that systemic infections can alter the intestinal microbiota [22], and current evidence suggests that there is a two-way interaction where alterations in gut microbiota affect infectious diseases, while infectious diseases in turn regulate the structure and function of gut microbiota [23–26]. However, there is little information on the intestinal microbiome of the hamster [27, 28], and to the best of our knowledge, the only relationship studied in this host species is between *L. infantum* infection and *Bifidobacterium* spp. and *Lactobacillus* spp. [29]. Thus, in the context of a wider project whose results will be published elsewhere, we have determined the 16S metagenomic composition of the intestinal microbiome of hamsters experimentally infected with *L. infantum*.

## Methods

### Experimental design

Syrian male hamsters (*Mesocricetus auratus*) (*n* = 14), aged 7–8 weeks, each weighing between 85 and 120 g, were purchased from Janvier Labs (Le Genest-Saint-Isle, France) and placed in quarantine. The animals were kept under observation and housed under a controlled temperature regimen and 12:12-h light:dark cycle (Instituto de Investigación Hospital 12 de Octubre, Madrid, Spain). They were provided with commercial pelleted food and water ad libitum. When the animals reached a weight of 120–140 g they were divided in a stratified manner (live weight [lw]) and inoculated with a canine-derived autochthonous strain of *L. infantum* (MCAN/ES/96/BCN150) (experimental group 3 [G3];* n* = 8) at 10^8^ promastigotes/hamster [19, 30] or maintained uninfected (control group 1 [G1]: *n* = 6). The animals were euthanized at approximately 19 weeks post inoculation (wpi). The size of the uninfected control group was kept to a minimum on ethical grounds and from prior experience with such control groups.

The lw was determined on day 0 (preinfection), 16 wpi and at the endpoint of the experiment. Blood samples were obtained from the cava vein under anaesthesia (isoflurane 2–4%) before infection and 16 wpi; the endpoint sample was obtained by intracardiac puncture. Sera from all the experimental animals were used to determine the infection status of the hamsters by the immunoglobulin G enzyme-linked immunosorbent immunoglobulin (IgG ELISA) using the standard indirect ELISA protocol from our laboratory. The 96-well ELISA plates (Nunc MaxiSorp™, Thermo Fisher Scientific, Waltham, MA, USA) were coated with 50 µl/well of 7.5 µg/ml soluble *L. infantum* antigen in HCO_3_^–^/CO_3_^–^ (4 °C, overnight). Sera samples were added to the wells at 1/100 dilution, 50 µl/well and incubated at 37 °C for 1 h. The secondary antibody was goat anti-hamster IgG (H+L)-HRP (Southern Biotech, Birmingham, AL, USA) (1/2000 dilution; incubated for 30 min at room temperature);* o*-phenylenediamine (1 mg/ml) (Sigma-Aldrich, St. Louis, MO, USA) + H_2_O_2_ (1/1000) solution were added (100 µl/well) and the reaction was stopped with the addition of 50 µl/well of H_2_SO_4_ (3 N). Absorbance at 492 nm was determined in a Multiskan™ GO Microplate Spectrophotometer (Thermo Fisher Scientific). ELISA results (optical density [OD]) were expressed as a percentage of the positive control serum. The OD cut-off (±) was established at mean preinfection values of +3 standard deviations (SD) (13.74%). All determinations were performed at least in triplicate.

### Genomic DNA extraction, 16S metagenome library construction and next-generation sequencing

Individual faecal samples were obtained at week 16 pi and week 18 pi by temporarily isolating individual animals and collecting faecal pellets immediately after dropping. At the end of the experiment (endpoint: 19 wpi), faecal samples were obtained from the rectum during the necropsy and stored at − 80 °C until processing. For the genomic analyses, five faecal samples from G1 (uninfected control hamsters) and four from G3 (*L. infantum*-infected hamsters) were randomly selected from each sampling time.

DNA extraction, construction of the next-generation sequencing (NGS) library and sequencing were carried out at the Unidad de Genómica, Complutense University of Madrid (Spain). Total DNA from hamster faecal samples was extracted with the DNeasy PowerLyser PowerSoil DNA Kit (Qiagen, Hilden, Germany) following the manufacturer’s instructions. DNA concentration was estimated using the Qubit 2.0 fluorimeter (Life Technologies™, Thermo Fisher Scientific). DNA libraries from each sample were prepared following the Illumina 16S Metagenomic Sequencing Library Preparation manual (Illumina, San Diego CA, USA). In brief, the V3–V4 region of the prokaryotic 16S ribosomal RNA (rRNA) was amplified for each sample with primers containing the 341F and 805R sequences and Illumina-specific adapters. In a second PCR amplification, two specific 8-nucleotide index and i5/i7 Illumina adapters were added to the previous amplicons. DNA libraries were checked with the Bioanalyzer 2100 platform (Agilent Technologies, Palo Alto, CA, USA). A library pool was prepared for sequencing by mixing equal amounts of the individual sample libraries, and then sequenced in the Illumina MiSeq benchtop sequencer with 2 × 300 reads using the 600 cycle MiSeq Reagent Kit v3 in accordance with the manufacturer’s recommended protocol.

### Sequence data analysis

The FASTQ files containing the sequencing reads were analysed using the CLC Genomics Workbench version 20.0.4 (QIAGEN Aarhus A/S, Aarhus, Denmark; http://www.qiagenbioinformatics.com). Sequence data were trimmed using 0.05 as a limit for quality scores, with 2 as the maximum number of ambiguities. The reads after trimming were analysed using the CLC Microbial Genomics Module version 20.1.1. The optional merge paired reads method was run with default settings (mismatch cost = 1; minimum score = 40; gap cost = 4; maximum unaligned end mismatch = 5). Sequence reads were clustered and chimeric sequences detected using an identity of 97% as the operational taxonomic unit (OTU) threshold. The reference OTU data used in the present study were downloaded from the SILVA database [31] v132 for 16S rRNA. Shannon’s diversity index was calculated considering the assigned species. The raw sequencing data were deposited in the NCBI Sequence Read Archive database (BioProject ID: PRJNA843999) (https://www.ncbi.nlm.nih.gov/bioproject/PRJNA843999).

### Statistical analysis

The experimental groups were included in a larger experiment, and the number of animals was selected to give a Z-power of 0.8 and a 95% level of significance. Unless otherwise stated, the numerical values are presented as the mean ± SD. Statistical analyses included parametric and non-parametric tests (1w- and 2w-analysis of variance [ANOVA], Mann–Whitney test, Student t-test), and the level of significance was set at *P* ≤ 0.05. The taxonomic comparison between the groups was performed with the differential abundance analysis tool from the CLC Microbial Genomics Module. The table of OTUs generated by the CLC Microbial Genomics Module from each microbiome classified at phylum, family or genus levels was used as the input. Unless otherwise stated, only changes of at least ± twofold (±) in the present taxa, and false discovery rates (FDR) with an adjusted *P*-value of ≤ 0.05, were considered as significant. The figures were prepared with GraphPad Prism 9.0 (GraphPad Software, San Diego, CA, USA) and Microsoft Excel (Microsoft Corp., Redmond, WA, USA).

## Results

### *Leishmania infantum* infection

None of the non-inoculated animals developed any cutaneous lesion throughout the experiment, whereas lesions were observed in five of the eight infected hamsters (62.5%). Lesions included dermatitis, exfoliative dermatitis and crusts in different locations (e.g. back, abdomen, legs, neck, inguinal and axillar regions, ears) and alopecia (chest, face, periorbitary). Inoculation with *L. infantum* did not cause significant lw loss, and hamsters reached comparable lw at week 16 irrespective of their infection status (161.85 ± 13.19 g [uninfected G1] vs 162.33 ± 11.15 g) [infected G3]).

**Fig. 1:**
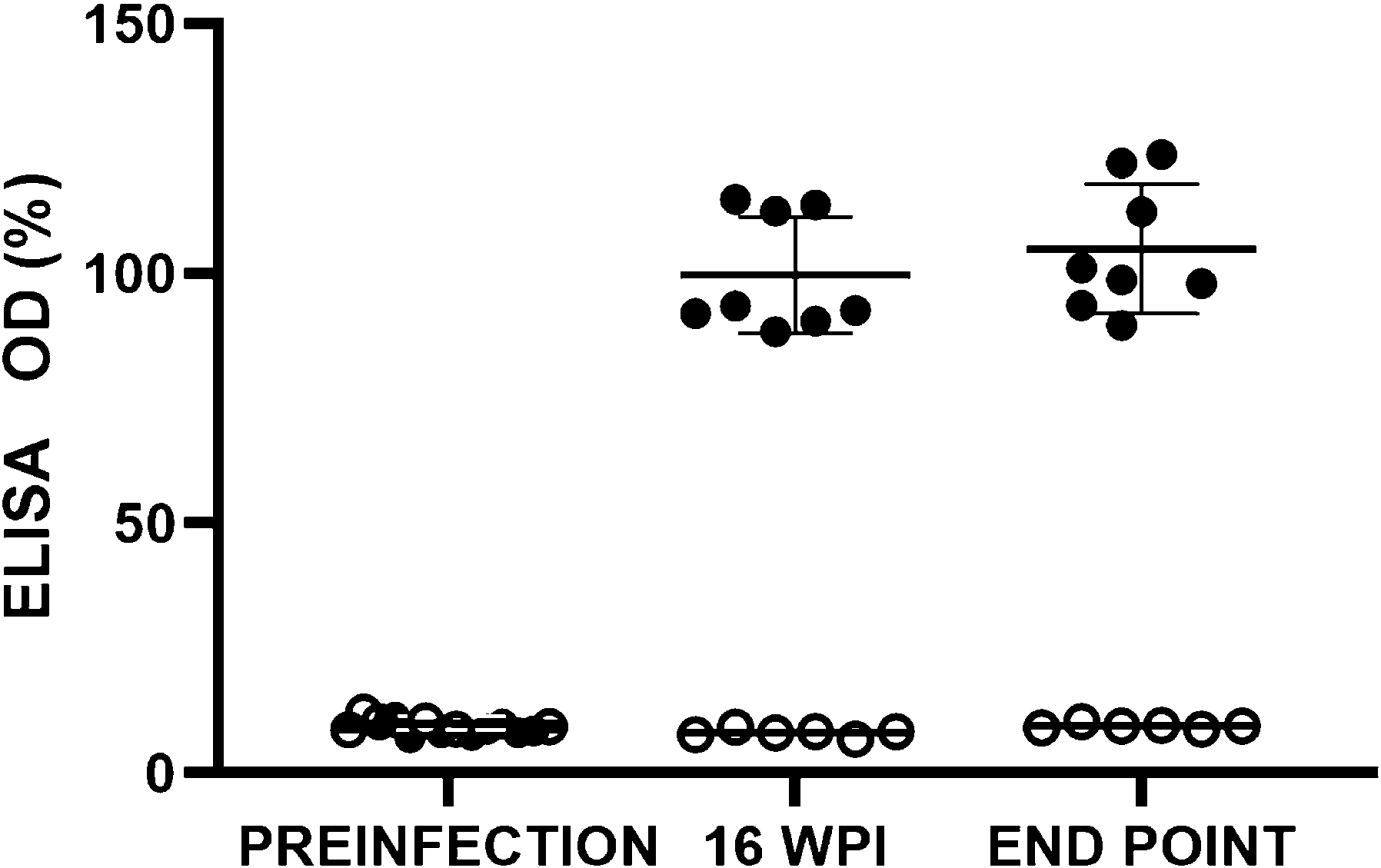
Serum-specific anti-*Leishmania* immunoglobulin G (IgG) response of inoculated (G3) (solid circles) and control (G1) (empty circles) hamsters throughout the experiment. ELISA values correspond to the percentage OD from the positive control sera. Serum IgG levels were determined before infection (preinfection), 16 WPI and at the endpoint of the experiment (19 WPI). Individual values, mean of the group and standard deviation (%) are given. ELISA, Enzyme-linked immunosorbent assay; OD, optical density; WPI, weeks post inoculation

The control animals (G1) showed no variation in the the serum-specific IgG response throughout the experiment, and their OD values were in all cases below the cut-off (Fig. 1). In the inoculated animals (G3), there was a notable increase in anti-*Leishmania* IgG levels at week 16 pi and 3 weeks later (i.e. endpoint: 19 weeks) when the inoculated hamsters attained an anti-*Leishmania* IgG level of 104.9 ± 12.9% of that of the positive control serum compared to the uninfected control group (9.4 ± 0.5%).

### Intestinal microbiome of the control hamsters

**Fig. 2:**
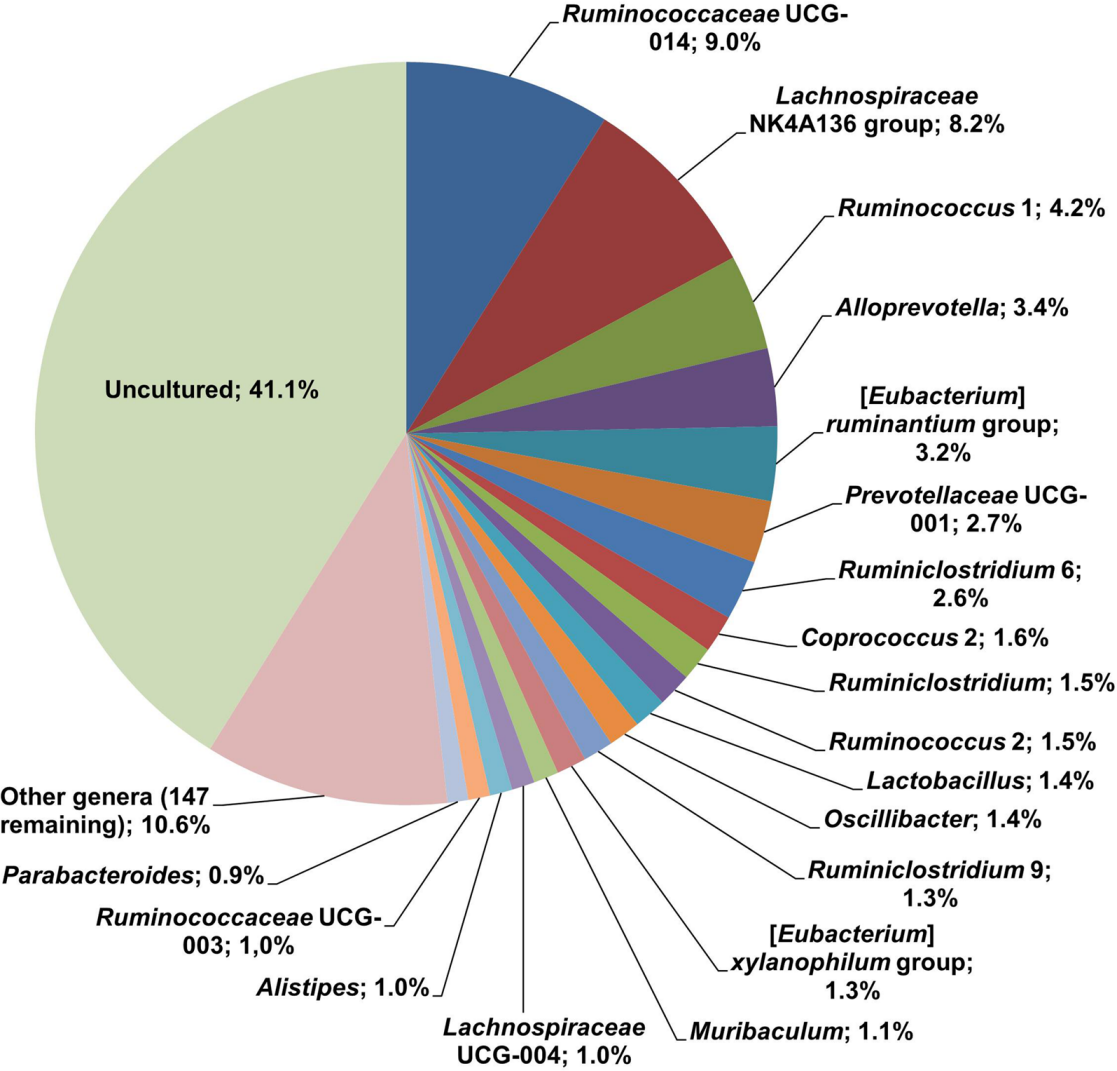
Dominant genera (20 most abundant) identified in the faecal samples obtained from the uninfected control hamsters (G1) at week 16 of the experiment (16 wpi). The values represent the mean relative abundance (%) in the samples analysed (*n* = 5)

The analysis of the predominant taxa (OTU) in the microbiome in the faecal samples obtained at week 16 yielded 17 phyla. The most abundant were *Firmicutes* and *Bacteriodetes*, representing an average of ≥ 90% of the OTUs, followed by *Verrucomicrobia* (1.7–< 0.1%), *Proteobacteria* (1.9–0.7%), *Cyanobacteria* (1.3–0.4%), *Patescibacteria* (1.4–0.1%), *Actinobacteria* (0.3–< 0.1%), *Tenericutes*, *Deferribacteres* and *Elusimicrobia*. The most represented families were: *Muribaculaceae* (*Bacteroidetes*), *Lachnospiraceae* (*Firmicutes*) and *Ruminococcaceae* (*Firmicutes*), which accounted for 70–80% of the total number (*n* = 78) of families; *Prevotellaceae* (Phy. *Bacteroidetes*), *Akkermansiaceae* (Phy. *Verrucomicrobia*) and *Lactobacillaceae* (Phy. *Firmicutes*) represented approximately 6–12% of families (Additional file 1: Table S1). A total of 169 genera were found, of which—in addition to the dominant uncultured bacterium (41.1 ± 6%)—the most abundant were the *Lachnospiraceae* NK4A136 group (*Firmicutes*, *Lachnospiraceae*), *Ruminococcaceae* UCG-014 (*Firmicutes*, *Ruminococcaceae*) and *Alloprevotella* (*Bacteroidetes*, *Prevotellaceae*), although there was ample representation of other genera (Fig. 2).

**Fig. 3:**
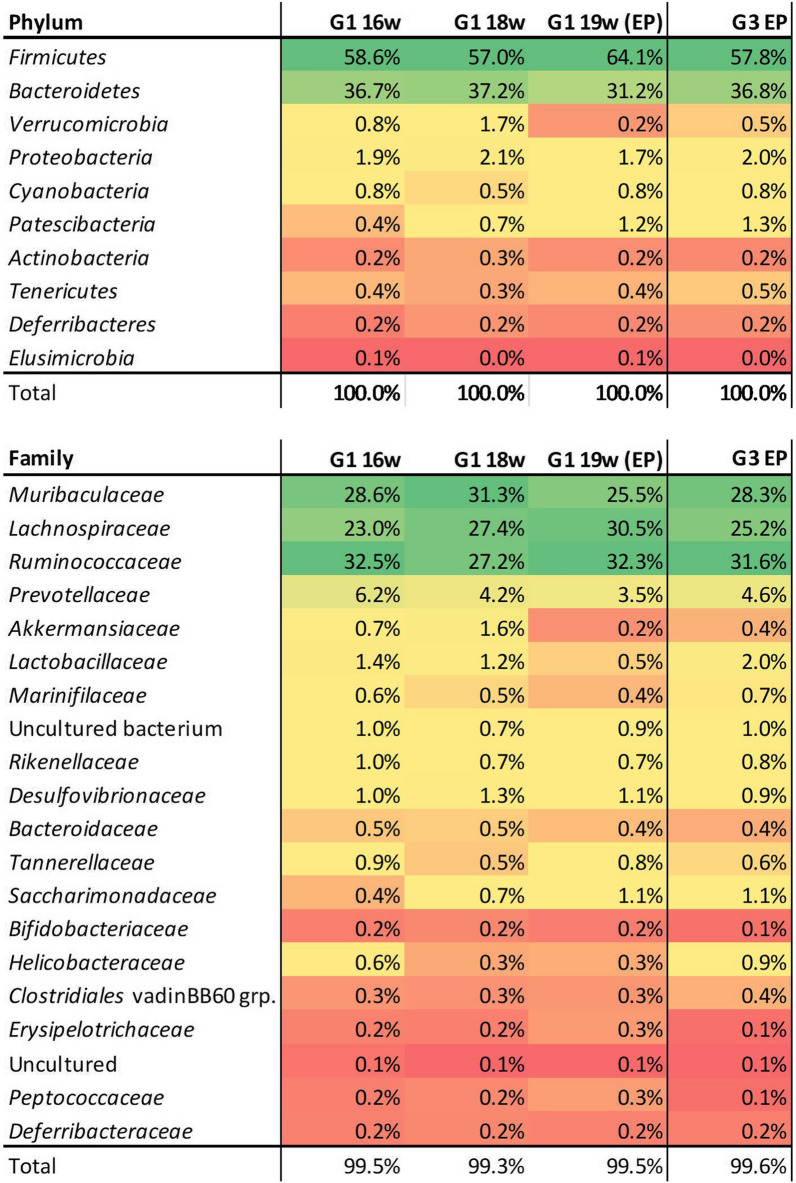
Relative abundance (% and colour scale) of the most represented phyla and families in the faecal samples from the uninfected hamsters (G1) throughout the experimental period, and from the infected hamsters (G3) at the endpoint. Values represent the mean relative abundance (%) in the samples (*n* = 5 for G1 samples;* n* = 4 for G3 samples). The colour scale (green to red) shows a graphic representation of the relative abundance within each group and sampling time. 16w, 16 weeks post inoculation; 18w, 18 weeks post inoculation; 19w (EP), 19 weeks post inoculation (endpoint)

The relative abundance of OTUs in the uninfected animals (G1) did not vary for the most prevalent taxa throughout the experimental period, and Shannon’s index suggested a similar diversity (16 weeks: 3.3; 18 weeks: 3.1; 19 weeks: 3.3). The microbiome analysis of the uninfected hamsters was therefore homogeneous in terms of the most represented phyla, most abundant families (Fig. 3) and the genera. The statistical analysis revealed no significant difference throughout the experiment.

### Effect of *L. infantum* infection on microbiome composition

No significant differences were found in the phyla at 16 wpi between the uninfected control hamsters (G1) and hamsters infected with *L. infantum* (G3) (G1 vs G3, *P* = 0.414), with OTUs of > 99.9% of all reads. This lack of significant differences was also observed 2 weeks later (G1 vs G3, FDR *P-*value = 0.196) and at the endpoint of the experiment (G1 vs G3, FDR *P-*value = 0.99) (Fig. 3). Despite the slightly higher value of the *Firmicutes*/*Bacteroidetes* (F/B) ratio found in infected animals (F/B = 2.47), the difference was not significant. Similar results were obtained for the families, considering the 30 most abundant (G1 vs G3, FDR *P-*value ≥ 0.99) for all the sampling times (16 weeks, 18 weeks and endpoint).

No significant differences were found in any of the time-matched samplings when the values of the 30 most abundant genera were compared (> 94% reads). Moreover, neither the phyla, families nor genera in the control group showed any significant variation between the three sampling times (G1: uninfected: 16 vs 18 weeks vs endpoint; G3: 16 vs 18 weeks vs endpoint).

**Fig. 4:**
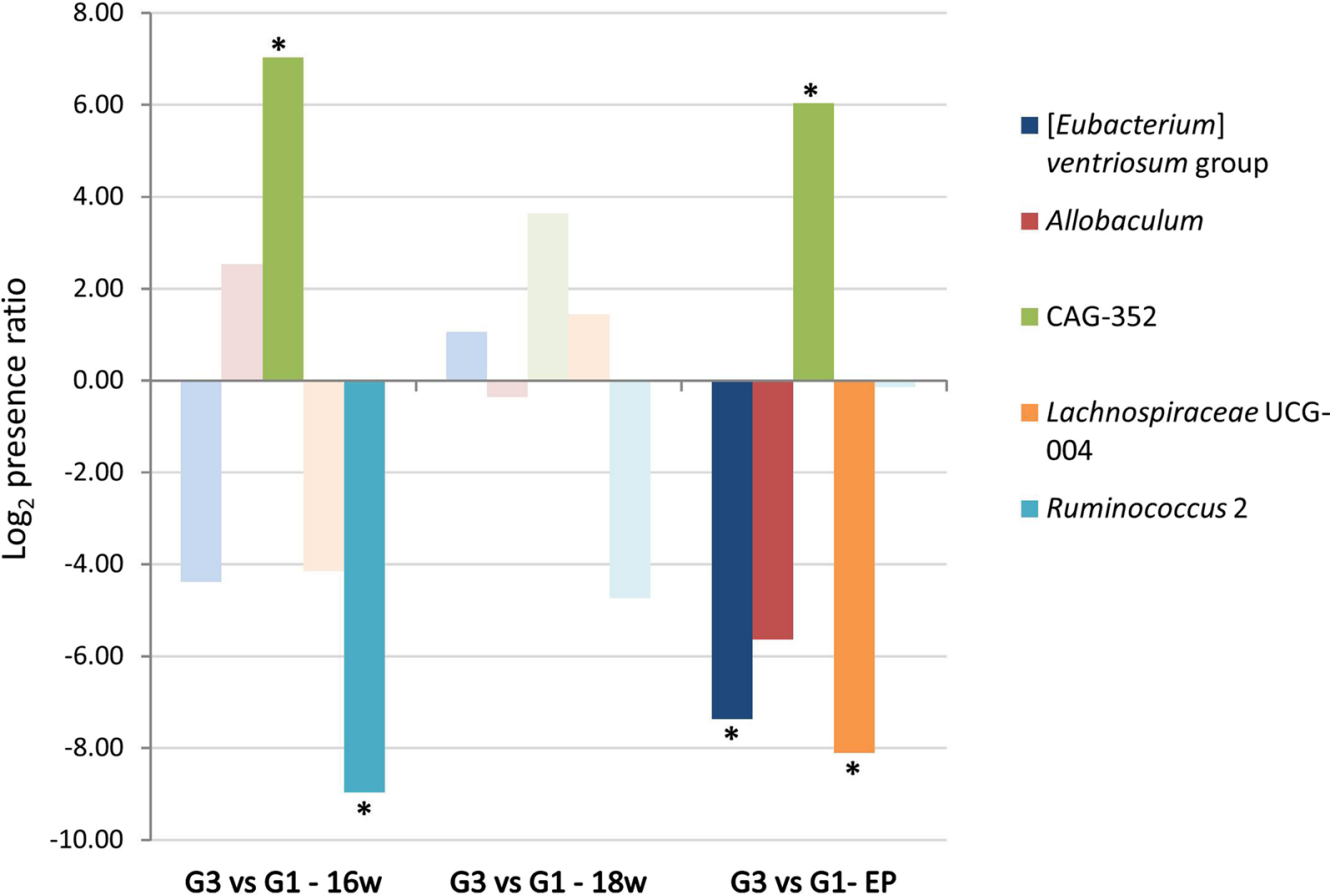
Comparison of genera abundance (log_2_) in the intestinal microbiome of hamsters infected with *L. infantum* (G3) and uninfected (G1) throughout the experiment. Asterisk indicates a statistically significant difference (false discovery rate [FDR] *P-*value < 0.05)

The taxonomic comparison (FDR analysis, FDR *P*-value) of the hamster groups showed no significant differences in phyla between the uninfected control animals (G1) and the *L. infantum*-infected hamsters at 16 weeks (G3); this lack of significance was maintained for the entire experiment (minimum FDR value in all comparisons = 0.0975). Similarly, there were no significant differences in the relative abundance of families in both groups (minimum FDR *P-*value in all comparisons = 0.1237) (Additional file 2: Table S2). However, there were variations in the relative abundance of the genera identified: CAG-352 (*Clostridia*, *Firmicutes*) significantly increased its presence at week 16 (FDR *P-*value = 0.0078), week 18 and at the endpoint of the experiment (week 19, FDR *P-*value = 0.0309), while in the final sampling, *Lachnospiraceae* UCG-004, [*Eubacterium*] *ventriosum* and the *Allobaculum* group showed a significantly lower abundance (FDR *P-*value = 0.0059, 0.0015 and 0.0314, respectively). Significant differences (FDR *P-*value = 3.51 × 10^–6^ in the relative abundance of *Ruminococcus* 2 were found between groups at week 16 but these progressively disappeared, and no differences were observed by the end of the experiment (FDR *P-*value > 0.05) (Fig. 4).

## Discussion

The course of leishmaniasis is dependent on the imbalance in the host’s immune response [13, 14], which in turn is related to the virulence of *Leishmania* and the presence of intercurrent infections, among other factors. In experimental infections in surrogate models, the dose administered has been shown to affect the infection outcome with different *Leishmania* spp. [32–34]. Experimental infections of VL in hamsters have been carried out with a variety of infective doses and inoculation procedures: intracardiac [15, 18, 35–37], intraperitoneal [36, 38] and intradermic) [36]. In our experiment, hamsters were inoculated by the retro-orbitary route with 10^8^ stationary phase promastigotes of *L. infantum*, since both infective dose and inoculation procedure elicit consistent infections in hamsters with the same *L. infantum* strain [39].

Under our experimental conditions, the inoculation of Syrian hamsters with *L. infantum* promastigotes provoked a steady rise in specific antibodies in all inoculated animals throughout the experiment, confirming the infectivity of the parasite strain and the efficiency of the inoculation procedure. The absence of weight loss and the moderate cutaneous alterations in the infected hamsters were comparable to previous findings under similar experimental conditions (age of animals, infective dose, *Leishmania* strain) [19]. Although most exploratory research on VL (e.g. immune response, pathology, vaccine candidates, chemotherapeutic lead compounds) is done in mice, hamster infections are long-lasting [15] and mimic the infections in the natural hosts (humans, dogs) more closely since they lack the nitric oxide response against *Leishmania* [16] and are considered to be a better model for VL [17–20]. The relationship between the intestinal microbiome and health is considered to be of paramount importance and has fuelled the exploration of its composition both in humans [21–24, 26, 40] and in dogs and cats [41–43]. While there is abundant information on the intestinal microbiome of mice [26, 44–46], very few studies have been conducted and published on hamsters. We found a higher diversity in the microbiome than previously reported for Syrian hamsters [47–49]. The most abundant phyla (*Firmicutes*, *Bacteriodetes*, *Verrucomicrobia*, *Proteobacteria*, *Saccharibacteria*) had previously been reported in this animal species, with the exception of a nutrition-focused study [48]. The relative abundance was variable, although the values for the main phyla were comparable to those of the most recent study [49]; the predominance of *Firmicutes* (94%) reported by Martínez et al. [47] could not be confirmed. In fact, the relative abundance of OTUs in our study in the case of phyla is in line with the findings reported in most animal species, including mice [46, 48], dogs and cats [41, 42, 50, 51] and humans [52, 53]. The high variability reported could be related to the strong influence of diet, genotype and environmental factors [44, 54], including housing conditions, which—together with the different methodologies used—would affect their inter-laboratory reproducibility. This is relevant since the results cannot be compared unless standardized designs and methods are used.

The interaction between several pathological conditions and the intestinal microbiome composition of humans [21, 24–26, 53] and other animals, including dogs and cats [41, 42, 51], has been explored. In the only study carried out in humans with VL, despite the reduction of *Ruminococcaceae* UCG-014 and *Gastranaerophilales* uncultured bacterium in VL patients (*n* = 23), the overall comparison (alpha and beta analyses) showed no variation in infected individuals [55]. Cross-sectional studies are hampered by individual variations (diet, habits), and data derived from such studies are thus difficult to interpret and extrapolate. In our study the hamsters were obtained from a standardized supplier and maintained throughout the experiment in the same animal facilities and under the same conditions; thus, the intergroup differences (uninfected vs *L. infantum*-infected animals) could be considered to be factual. In the only study carried out so far with *Leishmania*-infected hamsters under controlled conditions, no relationship was found between the abundance of intestinal *Bifidobacterium* spp. and *Lactobacillus* and infection with *L. infantum* despite the duration of the infection (up to 8 months) [29]. In the present experiment, we used a powerful analytical technique (16S) followed by a robust statistical analysis (FDR filtered *P*-value < 0.05), and found no major differences in the relative abundance of phyla and families in the intestinal microbiome; the variation observed was restricted to certain non-abundant genera (CAG-352, *Lachnospiraceae* UCG-004, [*Eubacterium*] *ventriosum* group and *Allobaculum*). *CAG-352* has been linked to human prostate cancer [56], and the role of the *Lachnospiraceae* family is far from clear [52, 57]. No mechanistic explanation is therefore available on the variation of these Clostridia (*Firmicutes*) in *L. infantum*-infected hamsters. Increases in butyrate-producing isolates of *Eubacterium* spp. have been related to higher body mass, which implies more efficient energy utilization [58]. However, in our case no significant differences were observed in the lw of the experimental animals. More recently this genus has also been implicated in the modulation of inflammation and the regulation of immune responses [59], and leishmaniasis outcomes are linked to an unbalanced host-parasite immune response [13, 14]. In our experiment the infected hamsters showed a 165-fold reduction in the [*Eubacterium*] *ventriosum* group compared to the uninfected animals (endpoint, week 19), accompanied by high levels of anti-*Leishmania* IgG antibodies. Although this relationship is suggestive, the number of experimental animals, duration of the infection and low relative abundance of the taxon (0.2% in healthy hamsters) do not support a causality relationship. More research is therefore required.

## Conclusions

In this study, Syrian hamsters were inoculated with a canine-derived *L infantum* strain and maintained for 19 weeks. The infection status of the animals was assessed by the presence of clinical signs and lesions and indirect IgG ELISA. The relationship between the intestinal microbiome (16S) and *L. infantum* infection in hamsters was studied for the first time. No major differences were found in higher taxa, and the actual significance of the slight variations found in non-abundant genera is unknown. Although we are aware of the limitations of the study (e.g. study duration, number of animals, co-abundance analysis and additional parasitological determinations) and the probable scarce translatability of microbiome results in rodents to the target hosts [54], the main conclusion is that no clear VL infection-intestinal microbiome axis has been identified. This finding should be confirmed in the future with chronically infected animals and by precisely determining the possible role of specific bacterial differences in the pathogenesis of VL.

## Supplementary Information

**Additional file 1: Table S1.** Identification and relative quantification of phyla, families and genera detected in individual microbiomes of G1-16w samples.

**Additional file 2: Table S2.** Changes in microbiota composition between G1 and G3 groups, expressed as log_2_ ratio, at the level of phylum, family and genus. The FDR *P*-value is indicated. 16w: 16 weeks post inoculation; 18w: 18 weeks post inoculation; EP: end point.

## Data Availability

All relevant data are given in the manuscript and Supplementary information. Materials, when available, can be requested to the authors.

## Abbreviations

FDR: False discovery rate
IgG ELISA: Immunoglobulin G enzyme-linked immunosorbent assay
lw: Live weight
NGS: Next-generation sequencing
OTU: Operational taxonomic unit
VL: Visceral leishmaniasis
wpi: weeks post inoculation
