# Supplementary material for: Leishmania infantum infection does not affect the main composition of the intestinal microbiome of the Syrian hamster
Source: Parasit Vectors. 2022 Dec 15;15:468. doi: 10.1186/s13071-022-05576-1 (PMC9753363; doi:10.1186/s13071-022-05576-1)
Supplement: Supplementary file 1 — Additional file 1: Table S1. Identification and relative quantification of phyla, families and genera detected in individual microbiomes of G1-16w samples. [file 13071_2022_5576_MOESM1_ESM.pdf]

Additional file 1. Table S1. Identification and relative quantification of Phyla, Families and Genera detected in individual microbiomes of G1-16w samples.

**Phylum**	**G1-16w 13**	**G1-16w 18**	**G1-16w 20**	**G1-16w 30**	**G1-16w 43**	**Average**
Firmicutes	49,7%	48,1%	78,3%	47,0%	65,5%	57,7%
Bacteroidetes	45,7%	46,8%	18,4%	48,7%	29,7%	37,9%
Proteobacteria	0,8%	0,9%	1,9%	1,0%	1,4%	1,2%
Cyanobacteria	0,8%	0,8%	0,4%	1,3%	0,8%	0,8%
Verrucomicrobia	1,7%	1,0%	0,0%	0,3%	0,5%	0,7%
Epsilonbacteraeota	0,2%	1,3%	0,3%	0,5%	0,7%	0,6%
Patescibacteria	0,3%	0,2%	0,1%	0,3%	0,9%	0,4%
Tenericutes	0,3%	0,3%	0,1%	0,5%	0,4%	0,3%
Actinobacteria	0,3%	0,4%	0,0%	0,3%	0,1%	0,2%
Deferribacteres	0,1%	0,2%	0,4%	0,0%	0,1%	0,2%
Elusimicrobia	0,0%	0,1%	0,0%	0,1%	0,0%	0,1%
Fusobacteria	0,0%	0,0%	0,0%	0,0%	0,0%	0,0%
Spirochaetes	0,0%	0,0%	0,0%	0,0%	0,0%	0,0%
Lentisphaerae	0,0%	0,0%	0,0%	0,0%	0,0%	0,0%
Acidobacteria	0,0%	0,0%	0,0%	0,0%	0,0%	0,0%
Planctomycetes	0,0%	0,0%	0,0%	0,0%	0,0%	0,0%
Chloroflexi	0,0%	0,0%	0,0%	0,0%	0,0%	0,0%
**Total general**	100,0%	100,0%	100,0%	100,0%	100,0%	100,0%

Family	G1-16w 13	G1-16w 18	G1-16w 20	G1-16w 30	G1-16w 43	Average
Ruminococcaceae	23,2%	32,0%	36,3%	26,2%	44,9%	32,5%
Muribaculaceae	30,1%	40,9%	14,4%	33,9%	23,8%	28,6%
Lachnospiraceae	24,5%	12,1%	41,3%	18,2%	18,8%	23,0%
Prevotellaceae	12,6%	3,7%	2,5%	8,5%	3,7%	6,2%
Lactobacillaceae	1,4%	3,1%	0,0%	1,7%	1,0%	1,4%
Rikenellaceae	0,9%	0,6%	0,4%	2,1%	1,1%	1,0%
**Uncultured bacterium**	1,0%	1,0%	0,5%	1,6%	1,1%	1,0%
Desulfovibrionaceae	0,6%	0,7%	1,8%	0,7%	1,3%	1,0%
Tannerellaceae	0,4%	0,5%	0,5%	2,6%	0,5%	0,9%
Akkermansiaceae	1,7%	1,0%	0,0%	0,3%	0,5%	0,7%
Marinifilaceae	1,2%	0,3%	0,4%	0,9%	0,3%	0,6%
Helicobacteraceae	0,2%	1,3%	0,3%	0,5%	0,7%	0,6%
Bacteroidaceae	0,5%	0,7%	0,2%	0,6%	0,3%	0,5%
Saccharimonadaceae	0,3%	0,2%	0,1%	0,3%	0,9%	0,4%
***Clostridiales* vadinBB60 group**	0,2%	0,0%	0,2%	0,3%	0,4%	0,2%
Erysipelotrichaceae	0,2%	0,4%	0,0%	0,2%	0,1%	0,2%
Deferribacteraceae	0,1%	0,2%	0,4%	0,0%	0,1%	0,2%
Peptococcaceae	0,1%	0,1%	0,3%	0,1%	0,1%	0,2%
Bifidobacteriaceae	0,3%	0,3%	0,0%	0,2%	0,0%	0,2%
**Uncultured**	0,1%	0,1%	0,0%	0,2%	0,0%	0,1%
**Family XIII**	0,1%	0,1%	0,1%	0,1%	0,1%	0,1%
Christensenellaceae	0,0%	0,1%	0,0%	0,2%	0,0%	0,1%
Elusimicrobiaceae	0,0%	0,1%	0,0%	0,1%	0,0%	0,1%
Eggerthellaceae	0,0%	0,1%	0,0%	0,0%	0,1%	0,0%
Flavobacteriaceae	0,0%	0,1%	0,0%	0,1%	0,0%	0,0%
Burkholderiaceae	0,0%	0,1%	0,0%	0,0%	0,0%	0,0%
**Uncultured rumen bacterium**	0,0%	0,0%	0,0%	0,1%	0,0%	0,0%
**Uncultured *Erysipelotrichaceae* bacterium**	0,0%	0,0%	0,0%	0,1%	0,0%	0,0%
Caldicoprobacteraceae	0,0%	0,0%	0,0%	0,0%	0,0%	0,0%
**Unidentified**	0,0%	0,0%	0,0%	0,0%	0,0%	0,0%
Enterobacteriaceae	0,0%	0,0%	0,0%	0,0%	0,0%	0,0%
Anaeroplasmataceae	0,0%	0,0%	0,0%	0,0%	0,1%	0,0%
Streptococcaceae	0,0%	0,0%	0,0%	0,0%	0,0%	0,0%
Defluviitaleaceae	0,0%	0,0%	0,0%	0,0%	0,0%	0,0%
Atopobiaceae	0,0%	0,0%	0,0%	0,0%	0,0%	0,0%
Fusobacteriaceae	0,0%	0,0%	0,0%	0,0%	0,0%	0,0%
Promicromonosporaceae	0,0%	0,0%	0,0%	0,0%	0,0%	0,0%
**Uncultured *Lachnospiraceae* bacterium**	0,0%	0,0%	0,0%	0,0%	0,0%	0,0%
Propionibacteriaceae	0,0%	0,0%	0,0%	0,0%	0,0%	0,0%
Moraxellaceae	0,0%	0,0%	0,0%	0,0%	0,0%	0,0%
***Blattella germanica* (German cockroach)**	0,0%	0,0%	0,0%	0,0%	0,0%	0,0%
Veillonellaceae	0,0%	0,0%	0,0%	0,0%	0,0%	0,0%
Halomonadaceae	0,0%	0,0%	0,0%	0,0%	0,0%	0,0%
Rubritaleaceae	0,0%	0,0%	0,0%	0,0%	0,0%	0,0%
Porphyromonadaceae	0,0%	0,0%	0,0%	0,0%	0,0%	0,0%
Spirochaetaceae	0,0%	0,0%	0,0%	0,0%	0,0%	0,0%
Corynebacteriaceae	0,0%	0,0%	0,0%	0,0%	0,0%	0,0%
Sphingomonadaceae	0,0%	0,0%	0,0%	0,0%	0,0%	0,0%
**VadinBE97**	0,0%	0,0%	0,0%	0,0%	0,0%	0,0%

**Gut metagenome**	0,0%	0,0%	0,0%	0,0%	0,0%	0,0%
Enterococcaceae	0,0%	0,0%	0,0%	0,0%	0,0%	0,0%
Nitriliruptoraceae	0,0%	0,0%	0,0%	0,0%	0,0%	0,0%
Pedosphaeraceae	0,0%	0,0%	0,0%	0,0%	0,0%	0,0%
Rhizobiaceae	0,0%	0,0%	0,0%	0,0%	0,0%	0,0%
Chthoniobacteraceae	0,0%	0,0%	0,0%	0,0%	0,0%	0,0%
Nocardioidaceae	0,0%	0,0%	0,0%	0,0%	0,0%	0,0%
Sporolactobacillaceae	0,0%	0,0%	0,0%	0,0%	0,0%	0,0%
Eubacteriaceae	0,0%	0,0%	0,0%	0,0%	0,0%	0,0%
Actinomycetaceae	0,0%	0,0%	0,0%	0,0%	0,0%	0,0%
Desulfobulbaceae	0,0%	0,0%	0,0%	0,0%	0,0%	0,0%
Xanthomonadaceae	0,0%	0,0%	0,0%	0,0%	0,0%	0,0%
Beijerinckiaceae	0,0%	0,0%	0,0%	0,0%	0,0%	0,0%
Rhodobacteraceae	0,0%	0,0%	0,0%	0,0%	0,0%	0,0%
Nicotiana benthamiana	0,0%	0,0%	0,0%	0,0%	0,0%	0,0%
Campylobacteraceae	0,0%	0,0%	0,0%	0,0%	0,0%	0,0%
Rubinisphaeraceae	0,0%	0,0%	0,0%	0,0%	0,0%	0,0%
Caulobacteraceae	0,0%	0,0%	0,0%	0,0%	0,0%	0,0%
***Candidatus Gastranaerophilales* bact. Zag_111**	0,0%	0,0%	0,0%	0,0%	0,0%	0,0%
Cyclobacteriaceae	0,0%	0,0%	0,0%	0,0%	0,0%	0,0%
Bacillaceae	0,0%	0,0%	0,0%	0,0%	0,0%	0,0%
Nodosilineaceae	0,0%	0,0%	0,0%	0,0%	0,0%	0,0%
Coriobacteriaceae	0,0%	0,0%	0,0%	0,0%	0,0%	0,0%
**Family XII**	0,0%	0,0%	0,0%	0,0%	0,0%	0,0%
Crocinitomicaceae	0,0%	0,0%	0,0%	0,0%	0,0%	0,0%
Pirellulaceae	0,0%	0,0%	0,0%	0,0%	0,0%	0,0%
Staphylococcaceae	0,0%	0,0%	0,0%	0,0%	0,0%	0,0%
Streptomycetaceae	0,0%	0,0%	0,0%	0,0%	0,0%	0,0%
**JG30-KF-CM45**	0,0%	0,0%	0,0%	0,0%	0,0%	0,0%
**Total general**	100,0%	100,0%	100,0%	100,0%	100,0%	100,0%

**Genus**	**G1-16w 13**	**G1-16w 18**	**G1-16w 20**	**G1-16w 30**	**G1-16w 43**	**Average**
**Uncultured bacterium**	29,4%	40,0%	14,2%	34,4%	23,6%	28,3%
**Uncultured**	6,1%	11,1%	23,8%	6,7%	16,3%	12,8%
***Ruminococcaceae* UCG-014**	7,8%	8,6%	2,0%	11,1%	15,3%	9,0%
***Lachnospiraceae* NK4A136 group**	10,9%	3,5%	14,9%	3,6%	8,0%	8,2%
***Ruminococcus* 1**	4,6%	8,4%	1,6%	4,5%	1,6%	4,2%
Alloprevotella	7,4%	0,4%	2,0%	4,5%	2,6%	3,4%
**[*Eubacterium*] *ruminantium* group**	5,9%	1,3%	3,5%	4,8%	0,7%	3,2%
***Prevotellaceae* UCG-001**	5,2%	3,2%	0,5%	3,7%	1,0%	2,7%
***Ruminiclostridium* 6**	1,5%	1,8%	4,7%	0,9%	4,1%	2,6%
***Coprococcus* 2**	0,4%	0,8%	3,9%	0,0%	2,9%	1,6%
Ruminiclostridium	1,5%	0,9%	2,8%	0,7%	1,6%	1,5%
***Ruminococcus* 2**	0,0%	0,0%	2,5%	0,2%	4,7%	1,5%
Lactobacillus	1,4%	3,1%	0,0%	1,7%	1,0%	1,4%
Oscillibacter	1,1%	0,5%	3,3%	1,1%	1,0%	1,4%
***Ruminiclostridium* 9**	1,0%	0,5%	3,1%	0,8%	1,2%	1,3%
**[*Eubacterium*] *xylanophilum* group**	0,9%	1,1%	3,0%	0,0%	1,6%	1,3%
Muribaculum	1,4%	1,2%	0,6%	0,9%	1,3%	1,1%
***Lachnospiraceae* UCG-004**	1,4%	0,0%	0,0%	3,4%	0,3%	1,0%
Alistipes	0,8%	0,6%	0,4%	2,1%	1,1%	1,0%
***Ruminococcaceae* UCG-003**	0,5%	1,0%	1,1%	1,0%	1,2%	1,0%
Parabacteroides	0,4%	0,5%	0,5%	2,6%	0,5%	0,9%
Akkermansia	1,7%	1,0%	0,0%	0,3%	0,5%	0,7%
**[*Eubacterium*] *coprostanoligenes* group**	0,7%	1,1%	0,1%	0,8%	0,3%	0,6%
**[*Eubacterium*] *ventriosum* group**	0,6%	0,0%	0,0%	2,3%	0,2%	0,6%
***Ruminococcaceae* UCG-010**	0,6%	0,9%	0,2%	0,8%	0,4%	0,6%
Helicobacter	0,2%	1,3%	0,3%	0,5%	0,7%	0,6%
**Uncultured *Bacteroidales* bacterium**	0,7%	0,7%	0,3%	0,7%	0,5%	0,6%
***Lachnospiraceae* UCG-006**	0,2%	0,2%	2,0%	0,2%	0,2%	0,5%
Bacteroides	0,5%	0,7%	0,2%	0,6%	0,3%	0,5%
Anaerosporobacter	0,9%	0,3%	1,1%	0,0%	0,0%	0,5%
Candidatus Saccharimonas	0,3%	0,2%	0,1%	0,3%	0,9%	0,4%
***Ruminiclostridium* 5**	0,3%	0,2%	1,0%	0,1%	0,3%	0,4%
***Ruminococcaceae* NK4A214 group**	0,2%	0,2%	0,4%	0,8%	0,3%	0,4%
**GCA-900066575**	0,2%	0,4%	0,6%	0,2%	0,4%	0,4%
Roseburia	0,2%	0,2%	0,8%	0,1%	0,2%	0,3%
Butyricimonas	0,2%	0,3%	0,3%	0,5%	0,2%	0,3%
Odoribacter	1,0%	0,0%	0,0%	0,4%	0,1%	0,3%
***Lachnospiraceae* UCG-001**	0,0%	0,1%	0,0%	0,0%	1,2%	0,3%
***Ruminococcaceae* UCG-005**	0,2%	0,2%	0,3%	0,4%	0,1%	0,2%
***Ruminococcaceae* UCG-013**	0,1%	0,2%	0,2%	0,2%	0,3%	0,2%
***Coprococcus* 3**	0,0%	0,6%	0,0%	0,2%	0,0%	0,2%
Mucispirillum	0,1%	0,2%	0,4%	0,0%	0,1%	0,2%
Lachnoclostridium	0,1%	0,1%	0,6%	0,0%	0,0%	0,2%
Bifidobacterium	0,2%	0,3%	0,0%	0,2%	0,0%	0,2%
***Lachnospiraceae* NC2004 group**	0,0%	0,1%	0,3%	0,1%	0,1%	0,1%
***Prevotellaceae* UCG-003**	0,1%	0,1%	0,0%	0,3%	0,1%	0,1%
Faecalibaculum	0,1%	0,3%	0,0%	0,0%	0,0%	0,1%
**Gut metagenome**	0,0%	0,1%	0,0%	0,2%	0,0%	0,1%
Desulfovibrio	0,0%	0,0%	0,3%	0,0%	0,0%	0,1%
***Lachnospiraceae* UCG-002**	0,0%	0,1%	0,2%	0,0%	0,0%	0,1%
***Christensenellaceae* R-7 group**	0,0%	0,1%	0,0%	0,2%	0,0%	0,1%
Butyricicoccus	0,0%	0,0%	0,2%	0,0%	0,0%	0,1%
***Ruminococcaceae* UCG-009**	0,0%	0,0%	0,1%	0,1%	0,0%	0,1%
Elusimicrobium	0,0%	0,1%	0,0%	0,1%	0,0%	0,1%
Pygmaiobacter	0,1%	0,0%	0,1%	0,0%	0,0%	0,1%
***Rikenellaceae* RC9 gut group**	0,1%	0,1%	0,0%	0,0%	0,0%	0,0%
***Ruminiclostridium* 1**	0,0%	0,1%	0,0%	0,1%	0,0%	0,0%
**UBA1819**	0,0%	0,0%	0,1%	0,0%	0,0%	0,0%
Oxalobacter	0,0%	0,1%	0,0%	0,0%	0,0%	0,0%

[*Eubacterium*] *oxidoreducens* group	0,0%	0,1%	0,0%	0,0%	0,0%	0,0%
Enterorhabdus	0,0%	0,0%	0,0%	0,0%	0,0%	0,0%
Acetatifactor	0,0%	0,0%	0,1%	0,0%	0,0%	0,0%
Anaerovorax	0,0%	0,0%	0,0%	0,0%	0,1%	0,0%
Tyzzerella	0,0%	0,0%	0,1%	0,0%	0,0%	0,0%
Lachnospiraceae UCG-008	0,0%	0,0%	0,0%	0,0%	0,0%	0,0%
Harryflintia	0,0%	0,0%	0,1%	0,0%	0,0%	0,0%
[*Ruminococcus*] *torques* group	0,0%	0,0%	0,1%	0,0%	0,0%	0,0%
Blautia	0,0%	0,0%	0,1%	0,0%	0,0%	0,0%
Dubosiella	0,0%	0,1%	0,0%	0,0%	0,0%	0,0%
**Family XIII UCG-001**	0,0%	0,0%	0,1%	0,0%	0,0%	0,0%
Caldicoprobacter	0,0%	0,0%	0,0%	0,0%	0,0%	0,0%
**GCA-900066225**	0,0%	0,0%	0,0%	0,0%	0,0%	0,0%
Peptococcus	0,0%	0,0%	0,1%	0,0%	0,0%	0,0%
[*Eubacterium*] *brachy* group	0,0%	0,0%	0,0%	0,0%	0,0%	0,0%
**Family XIII AD3011 group**	0,0%	0,0%	0,0%	0,0%	0,0%	0,0%
Anaerotruncus	0,0%	0,0%	0,1%	0,0%	0,0%	0,0%
Escherichia-Shigella	0,0%	0,0%	0,0%	0,0%	0,0%	0,0%
Lachnospiraceae FCS020 group	0,0%	0,0%	0,0%	0,0%	0,0%	0,0%
Anaeroplasma	0,0%	0,0%	0,0%	0,0%	0,1%	0,0%
**A2**	0,0%	0,0%	0,0%	0,0%	0,0%	0,0%
**Uncultured rumen bacterium**	0,0%	0,0%	0,0%	0,0%	0,0%	0,0%
Allobaculum	0,0%	0,0%	0,0%	0,1%	0,0%	0,0%
Streptococcus	0,0%	0,0%	0,0%	0,0%	0,0%	0,0%
Intestinimonas	0,0%	0,0%	0,0%	0,0%	0,0%	0,0%
Defluviitaleaceae UCG-011	0,0%	0,0%	0,0%	0,0%	0,0%	0,0%
Bilophila	0,0%	0,0%	0,0%	0,0%	0,0%	0,0%
**DNF00809**	0,0%	0,0%	0,0%	0,0%	0,0%	0,0%
Ruminococcaceae UCG-004	0,0%	0,0%	0,0%	0,0%	0,0%	0,0%
[*Eubacterium*] *nodatum* group	0,0%	0,0%	0,0%	0,0%	0,0%	0,0%
Uncultured *Clostridiales* bacterium	0,0%	0,0%	0,0%	0,0%	0,0%	0,0%
Coriobacteriaceae UCG-002	0,0%	0,0%	0,0%	0,0%	0,0%	0,0%
Papillibacter	0,0%	0,0%	0,0%	0,0%	0,0%	0,0%
Asaccharobacter	0,0%	0,0%	0,0%	0,0%	0,0%	0,0%
Anaerostipes	0,0%	0,0%	0,0%	0,0%	0,0%	0,0%
Acetitomaculum	0,0%	0,0%	0,0%	0,0%	0,0%	0,0%
Subdoligranulum	0,0%	0,0%	0,0%	0,0%	0,0%	0,0%
Dorea	0,0%	0,0%	0,0%	0,0%	0,0%	0,0%
Azospirillum sp. 47_25	0,0%	0,0%	0,0%	0,0%	0,0%	0,0%
Moryella	0,0%	0,0%	0,0%	0,0%	0,0%	0,0%
**Uncultured organism**	0,0%	0,0%	0,0%	0,0%	0,0%	0,0%
Oscillospira	0,0%	0,0%	0,0%	0,0%	0,0%	0,0%
Anaerocolumna	0,0%	0,0%	0,0%	0,0%	0,0%	0,0%
Marvinbryantia	0,0%	0,0%	0,0%	0,0%	0,0%	0,0%
Butyrivibrio	0,0%	0,0%	0,0%	0,0%	0,0%	0,0%
Fusobacterium	0,0%	0,0%	0,0%	0,0%	0,0%	0,0%
Cellulosimicrobium	0,0%	0,0%	0,0%	0,0%	0,0%	0,0%
Parasutterella	0,0%	0,0%	0,0%	0,0%	0,0%	0,0%
Alloscardovia	0,0%	0,0%	0,0%	0,0%	0,0%	0,0%
Turicibacter	0,0%	0,0%	0,0%	0,0%	0,0%	0,0%
Cutibacterium	0,0%	0,0%	0,0%	0,0%	0,0%	0,0%
Acinetobacter	0,0%	0,0%	0,0%	0,0%	0,0%	0,0%
**CAG-352**	0,0%	0,0%	0,0%	0,0%	0,0%	0,0%
Parvibacter	0,0%	0,0%	0,0%	0,0%	0,0%	0,0%
Halomonas	0,0%	0,0%	0,0%	0,0%	0,0%	0,0%
Morganella	0,0%	0,0%	0,0%	0,0%	0,0%	0,0%
Candidatus Soleaferrea	0,0%	0,0%	0,0%	0,0%	0,0%	0,0%
Megasphaera	0,0%	0,0%	0,0%	0,0%	0,0%	0,0%
Lachnospiraceae UCG-010	0,0%	0,0%	0,0%	0,0%	0,0%	0,0%
Luteolibacter	0,0%	0,0%	0,0%	0,0%	0,0%	0,0%

Porphyromonas	0,0%	0,0%	0,0%	0,0%	0,0%	0,0%
***Treponema* 2**	0,0%	0,0%	0,0%	0,0%	0,0%	0,0%
Negativibacillus	0,0%	0,0%	0,0%	0,0%	0,0%	0,0%
***Bacteroidales* bacterium 55_9**	0,0%	0,0%	0,0%	0,0%	0,0%	0,0%
Enterococcus	0,0%	0,0%	0,0%	0,0%	0,0%	0,0%
Eisenbergiella	0,0%	0,0%	0,0%	0,0%	0,0%	0,0%
**Uncultured *Verrucomicrobia* bacterium**	0,0%	0,0%	0,0%	0,0%	0,0%	0,0%
Ensifer	0,0%	0,0%	0,0%	0,0%	0,0%	0,0%
Candidatus Udaeobacter	0,0%	0,0%	0,0%	0,0%	0,0%	0,0%
**Uncultured *Acidobacteriaceae* bacterium**	0,0%	0,0%	0,0%	0,0%	0,0%	0,0%
Marmoricola	0,0%	0,0%	0,0%	0,0%	0,0%	0,0%
Proteus	0,0%	0,0%	0,0%	0,0%	0,0%	0,0%
Anaerofustis	0,0%	0,0%	0,0%	0,0%	0,0%	0,0%
Lawsonella	0,0%	0,0%	0,0%	0,0%	0,0%	0,0%
Actinomyces	0,0%	0,0%	0,0%	0,0%	0,0%	0,0%
**[*Desulfobacterium*] *catecholicum* group**	0,0%	0,0%	0,0%	0,0%	0,0%	0,0%
***Selenomonas* 3**	0,0%	0,0%	0,0%	0,0%	0,0%	0,0%
Sphingomonas	0,0%	0,0%	0,0%	0,0%	0,0%	0,0%
Salipaludibacillus	0,0%	0,0%	0,0%	0,0%	0,0%	0,0%
Salinarimonas	0,0%	0,0%	0,0%	0,0%	0,0%	0,0%
Nitriliruptor	0,0%	0,0%	0,0%	0,0%	0,0%	0,0%
Olsenella	0,0%	0,0%	0,0%	0,0%	0,0%	0,0%
Mogibacterium	0,0%	0,0%	0,0%	0,0%	0,0%	0,0%
***Lachnospiraceae* AC2044 group**	0,0%	0,0%	0,0%	0,0%	0,0%	0,0%
***Lachnospiraceae* UCG-009**	0,0%	0,0%	0,0%	0,0%	0,0%	0,0%
**SH-PL14**	0,0%	0,0%	0,0%	0,0%	0,0%	0,0%
Campylobacter	0,0%	0,0%	0,0%	0,0%	0,0%	0,0%
Rikenella	0,0%	0,0%	0,0%	0,0%	0,0%	0,0%
Porphyrobacter	0,0%	0,0%	0,0%	0,0%	0,0%	0,0%
Erythrobacter	0,0%	0,0%	0,0%	0,0%	0,0%	0,0%
Sutterella	0,0%	0,0%	0,0%	0,0%	0,0%	0,0%
Brevundimonas	0,0%	0,0%	0,0%	0,0%	0,0%	0,0%
***Nodosilinea* PCC-7104**	0,0%	0,0%	0,0%	0,0%	0,0%	0,0%
Egicoccus	0,0%	0,0%	0,0%	0,0%	0,0%	0,0%
Anaerobacillus	0,0%	0,0%	0,0%	0,0%	0,0%	0,0%
**Uncultured *Aquiflexum* sp.**	0,0%	0,0%	0,0%	0,0%	0,0%	0,0%
***Corynebacterium* 1**	0,0%	0,0%	0,0%	0,0%	0,0%	0,0%
Catabacter	0,0%	0,0%	0,0%	0,0%	0,0%	0,0%
Fluviicola	0,0%	0,0%	0,0%	0,0%	0,0%	0,0%
***Ruminococcaceae* UCG-007**	0,0%	0,0%	0,0%	0,0%	0,0%	0,0%
Alkalicoccus	0,0%	0,0%	0,0%	0,0%	0,0%	0,0%
Rhodopirellula	0,0%	0,0%	0,0%	0,0%	0,0%	0,0%
Collinsella	0,0%	0,0%	0,0%	0,0%	0,0%	0,0%
**ASF356**	0,0%	0,0%	0,0%	0,0%	0,0%	0,0%
Acidaminobacter	0,0%	0,0%	0,0%	0,0%	0,0%	0,0%
Natronohydrobacter	0,0%	0,0%	0,0%	0,0%	0,0%	0,0%
Staphylococcus	0,0%	0,0%	0,0%	0,0%	0,0%	0,0%
Streptomyces	0,0%	0,0%	0,0%	0,0%	0,0%	0,0%
Corynebacterium	0,0%	0,0%	0,0%	0,0%	0,0%	0,0%
***Tyzzerella* 4**	0,0%	0,0%	0,0%	0,0%	0,0%	0,0%
**Total general**	100,0%	100,0%	100,0%	100,0%	100,0%	100,0%
