# Supplementary material for: Leishmania infantum infection does not affect the main composition of the intestinal microbiome of the Syrian hamster
Source: Parasit Vectors. 2022 Dec 15;15:468. doi: 10.1186/s13071-022-05576-1 (PMC9753363; doi:10.1186/s13071-022-05576-1)
Supplement: Supplementary file 2 — Additional file 2: Table S2. Changes in microbiota composition between G1 and G3 groups, expressed as log2 ratio, at the level of phylum, family and genus. The FDR P-value is indicated. 16w: 16 weeks post inoculation; 18w: 18 weeks post inoculation; EP: end point. [file 13071_2022_5576_MOESM2_ESM.pdf]

Additional file 2. Table S2. Changes in microbiota composition between G1 and G3 groups, expressed as Log2 ratio, at the level of Phylum, Family and Genus. FDR p-value is indicated. 16w: 16 weeks post inoculation; 18w: 18 weeks post inoculation; EP: end point.

**Phylum**	**G3 vs G1 16w - Log_2 presence ratio**	**G3 vs G1 16w - FDR p-value**	**G3 vs G1 18w - Log_2 presence ratio**	**G3 vs G1 18w - FDR p-value**	**G3 vs G1 EP - Log_2 presence ratio**	**G3 vs G1 EP - FDR p-value**
Acidobacteria	-0,02	0,9902	-0,25	0,9708	-0,19	0,9884
Actinobacteria	-0,60	0,9849	-0,87	0,3543	-0,36	0,9884
Bacteroidetes	0,07	0,9849	0,03	0,9708	0,02	0,9884
BRC1	-0,21	0,9849	-0,25	0,9708	-0,19	0,9884
Chloroflexi	0,73	0,9849	-2,21	0,3543	-0,20	0,9884
Chrysiogenetes	-0,21	0,9849	-0,25	0,9708	-0,19	0,9884
Cyanobacteria	-0,26	0,9849	0,26	0,9708	-0,20	0,9884
Deferribacteres	0,18	0,9849	1,36	0,3543	-0,01	0,9884
Deinococcus-Thermus	-0,21	0,9849	-3,29	0,3543	-1,29	0,9884
Elusimicrobia	0,41	0,9849	3,94	0,0975	-1,13	0,9884
Epsilonbacteraeota	0,65	0,9849	1,21	0,3499	1,01	0,9884
Euryarchaeota	-0,21	0,9849	-0,25	0,9708	-2,35	0,9884
Firmicutes	-0,57	0,9849	-0,47	0,4738	-0,41	0,9884
Fusobacteria	-0,19	0,9849	-3,32	0,0975	-0,21	0,9884
Gemmatimonadetes	-0,21	0,9849	-0,25	0,9708	-0,19	0,9884
Lentisphaerae	2,30	0,7178	-0,02	0,9887	-3,20	0,6882
Patescibacteria	1,27	0,7178	0,05	0,9708	-0,02	0,9884
Planctomycetes	-0,74	0,9849	-0,25	0,9708	-0,79	0,9884
Proteobacteria	-0,77	0,9849	-1,27	0,3499	-0,83	0,9884
Spirochaetes	-1,23	0,9849	-1,79	0,3543	-0,10	0,9884
Synergistetes	-0,21	0,9849	-2,45	0,4738	-1,91	0,9884
Tenericutes	0,77	0,9849	0,59	0,7113	0,44	0,9884
Verrucomicrobia	0,08	0,9849	-0,42	0,9708	0,49	0,9884
WPS-2	-0,21	0,9849	-0,25	0,9708	-0,19	0,9884

Family	G3 vs G1 16w - Log₂ presence ratio	G3 vs G1 16w - FDR p-value	G3 vs G1 18w - Log₂ presence ratio	G3 vs G1 18w - FDR p-value	G3 vs G1 EP - Log₂ presence ratio	G3 vs G1 EP - FDR p-value
A4b	-0,51	0,8782	-2,84	0,6161	-0,11	0,9859
Acetobacteraceae	1,04	0,8782	-0,20	0,9455	-0,11	0,9859
Acidaminococcaceae	-0,51	0,8782	-0,20	0,9455	-0,11	0,9859
Actinomycetaceae	-0,40	0,8782	0,54	0,9455	-0,04	0,9900
Aerococcaceae	-0,51	0,8782	-0,20	0,9455	-0,12	0,9859
Aeromonadaceae	-0,51	0,8782	1,05	0,9455	-1,21	0,9859
Akkermansiaceae	-0,09	0,9385	-0,18	0,9455	0,42	0,9859
AKYG1722	-0,51	0,8782	-0,20	0,9455	-0,11	0,9859
Alteromonadaceae	-0,51	0,8782	-0,20	0,9455	-0,11	0,9859
Amoebophilaceae	-0,51	0,8782	-0,20	0,9455	-0,11	0,9859
Anaerolineaceae	1,04	0,8782	-0,20	0,9455	-0,11	0,9859
Anaeroplasmataceae	-1,23	0,8782	2,70	0,6161	-1,81	0,9859
Anaplasmataceae	-0,51	0,8782	-0,20	0,9455	-0,11	0,9859
Atopobiaceae	0,56	0,8782	-1,01	0,8587	0,75	0,9859
Bacillaceae	-1,90	0,8782	-4,51	0,2702	-2,06	0,9859
Bacteriovoracaceae	-0,51	0,8782	-0,20	0,9455	-0,11	0,9859
Bacteroidaceae	-0,56	0,8782	0,28	0,9455	-0,21	0,9859
Bacteroidales Incertae Sedis	-0,51	0,8782	1,71	0,9368	-1,83	0,9859
Bacteroidetes BD2-2	2,41	0,8782	-5,09	0,2604	-2,10	0,9859
Beggiatoaceae	-0,51	0,8782	-1,66	0,9455	-1,92	0,9859
Beijerinckiaceae	-0,89	0,8782	-4,54	0,2146	-1,40	0,9859
Bifidobacteriaceae	-0,87	0,8782	-0,43	0,9455	-0,44	0,9859
Blattella germanica (German cockroach)	-4,25	0,8782	1,19	0,9368	-4,40	0,9859
Bradymonadaceae	1,04	0,8782	-3,23	0,4847	-0,11	0,9859
Burkholderiaceae	-0,50	0,8782	-0,35	0,9455	-0,52	0,9859
Caldicoprobacteraceae	-0,77	0,8782	2,50	0,2146	0,64	0,9859
Campylobacteraceae	-0,35	0,8782	-0,20	0,9455	-0,11	0,9859
Candidate division SR1 bacterium taxon 345	-0,51	0,8782	-1,68	0,9368	1,09	0,9859
Candidatus Gastranaerophilales bacterium Zag_111	-0,35	0,8782	-0,20	0,9455	-0,11	0,9859
Cardiobacteriaceae	-0,51	0,8782	-0,20	0,9455	-0,11	0,9859
Carnobacteriaceae	-0,51	0,8782	-3,23	0,4847	-1,83	0,9859
Carya cathayensis	-0,51	0,8782	-0,20	0,9455	-0,11	0,9859
Caulobacteraceae	0,57	0,8782	-3,11	0,6161	-1,23	0,9859
Cellulomonadaceae	-0,51	0,8782	-0,20	0,9455	-0,11	0,9859
Chara vulgaris	-0,51	0,8782	-2,40	0,6691	-0,11	0,9859
Chitinophagaceae	-0,51	0,8782	-2,40	0,6691	-0,11	0,9859
Chloroflexaceae	-0,51	0,8782	-0,20	0,9455	-0,11	0,9859
Christensenellaceae	-0,19	0,8782	1,00	0,4875	-0,14	0,9859
Chromatiaceae	-0,51	0,8782	-4,08	0,3847	-0,12	0,9859
Chroococcidiopsaceae	-0,51	0,8782	-0,20	0,9455	-0,12	0,9859
Chrysiogenaceae	-0,51	0,8782	-0,20	0,9455	-0,11	0,9859
Chthoniobacteraceae	-2,59	0,8782	-0,20	0,9455	-0,11	0,9859
Clostridiaceae 1	-0,51	0,8782	-1,66	0,9455	1,70	0,9859
Clostridiaceae 2	-0,51	0,8782	-0,20	0,9455	-0,11	0,9859
Clostridiaceae 4	-0,51	0,8782	-0,20	0,9455	-0,11	0,9859
Clostridiales vadinBB60 group	-0,57	0,8782	-0,78	0,8728	0,47	0,9859
Coriobacteriaceae	-1,89	0,8782	1,05	0,9455	-1,21	0,9859
Coriobacteriales Incertae Sedis	1,08	0,8782	-2,42	0,7110	-0,66	0,9859
Corynebacteriaceae	-1,13	0,8782	-2,40	0,6691	1,41	0,9859
Crocinitomicaceae	0,95	0,8782	-4,34	0,2604	-0,11	0,9859
Cryomorphaceae	-0,51	0,8782	-0,20	0,9455	-0,11	0,9859
Cyclobacteriaceae	-0,44	0,8782	-4,60	0,3558	-2,94	0,9859
Deferribacteraceae	-0,22	0,8782	2,09	0,2604	0,03	0,9869
Defluviitaleaceae	1,44	0,8782	1,26	0,6691	1,48	0,9859
Desulfobacteraceae	-0,51	0,8782	-2,40	0,6691	-1,21	0,9859
Desulfobulbaceae	-2,55	0,8782	-3,46	0,4847	-2,33	0,9859
Desulfovibrionaceae	-1,46	0,8782	-1,59	0,4662	-0,97	0,9859
DEV007	1,04	0,8782	-1,68	0,9368	-0,11	0,9859
Devosiaceae	-0,51	0,8782	-0,20	0,9455	-0,11	0,9859

Dietziaceae	2,61	0,8782	-2,40	0,6691	-0,11	0,9859
Ectothiorhodospiraceae	1,03	0,8782	-3,48	0,4666	-0,11	0,9859
Eggerthellaceae	-0,41	0,8782	-1,05	0,5134	-0,27	0,9859
Elusimicrobiaceae	0,10	0,9439	4,20	0,1237	-1,18	0,9859
Endomicrobiaceae	-0,51	0,8782	-0,20	0,9455	-0,11	0,9859
Enterobacteriaceae	-3,63	0,8782	1,09	0,9368	-3,47	0,9859
Enterococcaceae	2,22	0,8782	0,96	0,9368	-1,75	0,9859
Env.OPS 17	-0,51	0,8782	-0,20	0,9455	-0,11	0,9859
Erysipelotrichaceae	0,26	0,8782	0,71	0,8638	-1,32	0,9859
Eubacteriaceae	-0,93	0,8782	0,23	0,9455	-0,21	0,9859
Euglena deses	-0,51	0,8782	-2,40	0,6691	-0,11	0,9859
Euzebyaceae	-0,51	0,8782	-0,20	0,9455	-0,11	0,9859
Family XI-01	-0,51	0,8782	-0,20	0,9455	-0,11	0,9859
Family XI-02	-0,51	0,8782	-2,04	0,5461	1,11	0,9859
Family XII	-1,89	0,8782	-0,20	0,9455	-0,11	0,9859
Family XIII	-0,88	0,8782	-1,70	0,2604	-0,83	0,9859
Fistulifera solaris	-0,51	0,8782	-0,20	0,9455	-0,11	0,9859
Flavobacteriaceae	-1,36	0,8782	0,54	0,9455	-0,13	0,9859
Fusobacteriaceae	-0,40	0,8782	-2,21	0,4662	-0,14	0,9859
Geminicoccaceae	-0,51	0,8782	-0,20	0,9455	-0,11	0,9859
Gemmatimonadaceae	-0,51	0,8782	-0,20	0,9455	-0,11	0,9859
Geobacteraceae	-0,51	0,8782	-2,40	0,6691	-0,11	0,9859
Gerbera hybrid cultivar	1,04	0,8782	-0,20	0,9455	-0,11	0,9859
Gracilibacteraceae	-0,51	0,8782	-0,20	0,9455	-0,11	0,9859
Gut metagenome-01	-0,30	0,8782	-0,20	0,9455	-0,11	0,9859
Gut metagenome-02	1,77	0,8782	-0,20	0,9455	-1,83	0,9859
GWF2-29-10	-0,51	0,8782	-0,20	0,9455	-0,11	0,9859
Halieaceae	-0,51	0,8782	-0,20	0,9455	-0,11	0,9859
Halomonadaceae	-3,92	0,8782	-5,56	0,2204	-3,49	0,9859
Haloplasmataceae	-0,51	0,8782	-0,20	0,9455	-0,11	0,9859
Helicobacteraceae	0,64	0,8782	1,16	0,4847	0,72	0,9859
Hydrothermal vent metagenome	-0,51	0,8782	-0,20	0,9455	-0,11	0,9859
Hymenobacteraceae	-0,51	0,8782	-1,68	0,9368	-1,21	0,9859
Hyphomonadaceae	-0,51	0,8782	-0,20	0,9455	-0,11	0,9859
Iamiaceae	-0,51	0,8782	-0,20	0,9455	-0,11	0,9859
Idiomarinaceae	-0,51	0,8782	-2,40	0,6691	-0,11	0,9859
Ilumatobacteraceae	-0,51	0,8782	-4,21	0,3769	-2,88	0,9859
Inquilinaceae	-0,51	0,8782	-0,20	0,9455	-0,11	0,9859
Intrasporangiaceae	-0,51	0,8782	-0,20	0,9455	-1,83	0,9859
Isosphaeraceae	-0,51	0,8782	-0,20	0,9455	1,09	0,9859
JG30-KF-CM45	-0,33	0,8782	-0,83	0,9455	-0,11	0,9859
Kryptoperidinium foliaceum	-0,51	0,8782	-1,68	0,9368	-0,11	0,9859
Lachnospiraceae	-1,08	0,8782	-0,81	0,7512	-0,69	0,9859
Lactobacillaceae	-0,84	0,8782	0,94	0,9312	2,01	0,9859
Legionellaceae	-0,51	0,8782	-0,20	0,9455	-0,11	0,9859
Lentimicrobiaceae	-0,51	0,8782	-0,20	0,9455	-0,11	0,9859
Leptotrichiaceae	-0,51	0,8782	-2,88	0,5736	-0,11	0,9859
Marinifilaceae	0,61	0,8782	-0,84	0,8638	0,90	0,9859
Marinilabiliaceae	-0,51	0,8782	-0,20	0,9455	-0,11	0,9859
Metagenome-01	-0,51	0,8782	-2,40	0,6691	-0,11	0,9859
Metagenome-02	-0,51	0,8782	-0,20	0,9455	-0,11	0,9859
Methanobacteriaceae	-0,51	0,8782	-0,20	0,9455	-2,25	0,9859
Methanosarcinaceae	-0,51	0,8782	-0,20	0,9455	-0,11	0,9859
Methylophilaceae	-0,51	0,8782	-0,20	0,9455	-0,11	0,9859
Microbacteriaceae	-0,51	0,8782	-2,40	0,6691	-0,11	0,9859
Micrococcaceae	-0,51	0,8782	-4,71	0,2604	-2,32	0,9859
Microtrichaceae	-0,51	0,8782	-0,20	0,9455	-0,11	0,9859
Mitochondria	1,04	0,8782	-0,20	0,9455	-0,01	0,9970
ML635J-40 aquatic group	-0,51	0,8782	-3,91	0,3847	-3,73	0,9859
Moraxellaceae	-1,97	0,8782	-4,54	0,2604	-1,05	0,9859

Muribaculaceae	-0,08	0,8782	-0,13	0,9455	0,00	0,9970
MWH-CFBk5	-0,51	0,8782	-1,68	0,9368	-0,11	0,9859
Mycoplasmataceae	-0,51	0,8782	-2,40	0,6691	-0,11	0,9859
Nannocystaceae	-0,51	0,8782	-0,20	0,9455	-1,21	0,9859
Nicotiana benthamiana	-1,89	0,8782	-0,20	0,9455	-0,11	0,9859
Nitriliruptoraceae	-1,41	0,8782	-3,56	0,4875	-2,27	0,9859
Nitrincolaceae	-0,51	0,8782	-0,20	0,9455	-1,21	0,9859
Nitrosomonadaceae	-0,51	0,8782	-0,20	0,9455	-0,11	0,9859
Nocardioidaceae	-2,60	0,8782	-0,20	0,9455	-0,11	0,9859
Nodosilineaceae	0,69	0,8782	-4,82	0,2719	-2,84	0,9859
Nostocaceae	-0,51	0,8782	-1,68	0,9368	1,74	0,9859
NS9 marine group	-0,51	0,8782	-2,40	0,6691	-0,11	0,9859
Opitutaceae	-0,51	0,8782	-1,68	0,9368	-0,11	0,9859
Oscillatoriaceae	-0,51	0,8782	-0,64	0,9455	-1,78	0,9859
Paludibacteraceae	-0,51	0,8782	-0,44	0,9455	-0,11	0,9859
Pedosphaeraceae	-2,59	0,8782	-0,20	0,9455	-0,11	0,9859
Peptococcaceae	-1,83	0,8782	-2,52	0,1514	-2,19	0,9859
Peptostreptococcaceae	-0,51	0,8782	-3,72	0,3905	-0,11	0,9859
Pinus sylvestris (Scots pine)	1,03	0,8782	1,74	0,9368	-1,23	0,9859
Pirellulaceae	-1,80	0,8782	-0,20	0,9455	-1,98	0,9859
Planococcaceae	2,07	0,8782	-5,37	0,2204	-0,13	0,9859
Planoglabratella opercularis	-0,51	0,8782	-0,20	0,9455	-0,11	0,9859
Porphyromonadaceae	-1,08	0,8782	-5,35	0,2146	-1,29	0,9859
Prevotellaceae	-0,24	0,8782	0,39	0,9455	0,25	0,9859
Prolixibacteraceae	-0,51	0,8782	-4,01	0,3769	-0,11	0,9859
Promicromonosporaceae	-0,94	0,8782	-1,00	0,9455	-1,84	0,9859
Propionibacteriaceae	-1,07	0,8782	-1,43	0,7512	-1,67	0,9859
Pseudohongiellaceae	-0,51	0,8782	-0,20	0,9455	-1,21	0,9859
Pseudomonadaceae	-0,51	0,8782	-3,74	0,4241	-0,12	0,9859
Puniceispirillales Incertae Sedis	-0,51	0,8782	-0,20	0,9455	-0,11	0,9859
Rhizobiaceae	-2,84	0,8782	-3,84	0,4398	-1,78	0,9859
Rhodanobacteraceae	-0,51	0,8782	-2,40	0,6691	-0,11	0,9859
Rhodobacteraceae	1,38	0,8782	-6,81	0,1237	-2,38	0,9859
Rhodocyclaceae	0,99	0,8782	-2,20	0,6691	-1,36	0,9859
Rikenellaceae	0,28	0,8782	0,83	0,6691	-0,11	0,9859
Rubinisphaeraceae	-1,89	0,8782	-0,20	0,9455	-0,11	0,9859
Rubritaleaceae	-3,28	0,8782	0,89	0,9455	-2,45	0,9859
Rubrobacteriaceae	-0,51	0,8782	-0,20	0,9455	-0,11	0,9859
Ruminococcaceae	-0,73	0,8782	-0,45	0,9368	-0,25	0,9859
Saccharimonadaceae	1,01	0,8782	0,04	0,9536	-0,05	0,9859
Sandaracinaceae	-0,51	0,8782	-0,20	0,9455	-0,11	0,9859
Sanguibacteraceae	-0,51	0,8782	-0,20	0,9455	-0,11	0,9859
Saprospiraceae	-0,51	0,8782	-0,21	0,9455	-1,25	0,9859
SB-5	-0,51	0,8782	-1,68	0,9368	-0,11	0,9859
SM2D12	-0,51	0,8782	-0,20	0,9455	-0,11	0,9859
Solibacteraceae (Subgroup 3)	1,77	0,8782	-0,20	0,9455	-0,11	0,9859
Sphingomonadaceae	-1,62	0,8782	-1,92	0,6691	-1,30	0,9859
Spirochaetaceae	-1,51	0,8782	-1,61	0,6161	0,01	0,9970
Spirogyra pratensis	-0,51	0,8782	-2,51	0,7185	-0,12	0,9859
Spirosomaceae	0,98	0,8782	-3,33	0,5461	-1,33	0,9859
Sporolactobacillaceae	-0,17	0,9338	-4,50	0,3769	-2,45	0,9859
Staphylococcaceae	0,38	0,8782	0,24	0,9455	-0,48	0,9859
Streptococcaceae	-1,25	0,8782	0,76	0,9455	-2,31	0,9859
Streptomycetaceae	-1,89	0,8782	-1,68	0,9368	-0,11	0,9859
Synergistaceae	-0,51	0,8782	-2,40	0,6691	-1,83	0,9859
Tannerellaceae	-0,92	0,8782	0,70	0,8609	-0,64	0,9859
Thiovulaceae	-0,51	0,8782	-0,20	0,9455	-0,11	0,9859
Trueperaceae	-0,51	0,8782	-3,23	0,4847	-1,21	0,9859
Uncultured bacterium-01	-0,51	0,8782	-0,20	0,9455	-2,26	0,9859
Uncultured bacterium-02	-0,51	0,8782	-0,20	0,9455	-0,11	0,9859

Uncultured bacterium-03	-0,51	0,8782	-0,20	0,9455	-0,11	0,9859
Uncultured bacterium-04	-0,62	0,8782	0,13	0,9455	-0,19	0,9859
Uncultured bacterium-05	1,77	0,8782	1,05	0,9455	-1,21	0,9859
Uncultured bacterium-06	1,77	0,8782	-0,20	0,9455	-0,11	0,9859
Uncultured bacterium-07	-0,51	0,8782	-0,20	0,9455	-0,11	0,9859
Uncultured bacterium-08	-0,51	0,8782	-1,68	0,9368	-1,21	0,9859
Uncultured bacterium-09	-0,51	0,8782	-2,40	0,6691	-0,11	0,9859
Uncultured bacterium-10	-0,51	0,8782	-1,68	0,9368	-1,21	0,9859
Uncultured bacterium-11	-0,51	0,8782	-0,46	0,9455	-2,35	0,9859
Uncultured bacterium-12	-0,51	0,8782	-0,20	0,9455	-0,11	0,9859
Uncultured bacterium-13	-0,51	0,8782	-0,20	0,9455	-0,11	0,9859
Uncultured bacterium-14	-0,51	0,8782	-0,20	0,9455	-0,11	0,9859
Uncultured bacterium-15	0,64	0,8782	0,65	0,9352	0,51	0,9859
Uncultured candidate division SR1 bacterium	-0,51	0,8782	-0,20	0,9455	-0,11	0,9859
Uncultured cyanobacterium	-0,51	0,8782	-0,20	0,9455	-0,11	0,9859
Uncultured *Erysipelotrichaceae* bacterium	-8,19	0,2038	8,23	0,1237	-2,11	0,9859
Uncultured eukaryote	0,97	0,8782	-3,66	0,4847	-0,30	0,9859
Uncultured *Lachnospiraceae* bacterium	1,15	0,8782	3,75	0,1237	2,00	0,9859
Uncultured *Microgenomates* group bacterium	-0,51	0,8782	-0,20	0,9455	-0,11	0,9859
Uncultured phototrophic eukaryote	5,07	0,8782	-0,22	0,9455	3,47	0,9859
Uncultured prokaryote	-0,51	0,8782	-0,20	0,9455	-0,11	0,9859
Uncultured rumen bacterium-01	1,27	0,8782	1,89	0,6691	-2,53	0,9859
Uncultured rumen bacterium-02	1,10	0,8782	-2,41	0,3723	-0,27	0,9859
Uncultured-01	-2,59	0,8782	-0,20	0,9455	-0,11	0,9859
Uncultured-02	-1,21	0,8782	-2,07	0,4847	1,49	0,9859
Uncultured-03	1,04	0,8782	-0,20	0,9455	1,09	0,9859
Uncultured-04	0,62	0,8782	1,30	0,5134	-0,37	0,9859
Unidentified	0,56	0,8782	0,83	0,8638	0,66	0,9859
Unknown Family-01	-0,51	0,8782	-0,20	0,9455	-0,11	0,9859
Unknown Family-02	-0,51	0,8782	-0,20	0,9455	-0,11	0,9859
VadinBE97	2,09	0,8782	0,17	0,9455	-2,99	0,9859
Veillonellaceae	-0,08	0,9515	-0,66	0,9455	-2,39	0,9859
Verrucomicrobiaceae	-0,51	0,8782	-2,40	0,7185	-1,90	0,9859
Victivallaceae	-0,51	0,8782	-0,20	0,9455	-0,11	0,9859
WD2101 soil group	-0,51	0,8782	-0,20	0,9455	-1,83	0,9859
Weeksellaceae	-0,51	0,8782	-0,20	0,9455	-1,83	0,9859
Woeseiaceae	-0,51	0,8782	-1,66	0,9455	-0,12	0,9859
Xanthobacteraceae	-0,51	0,8782	-0,20	0,9455	-0,11	0,9859
Xanthomonadaceae	-1,01	0,8782	-3,27	0,4140	-3,30	0,9859

Genus	G3 vs G1 16w - Log_2 presence ratio	G3 vs G1 16w - FDR p-value	G3 vs G1 18w - Log_2 presence ratio	G3 vs G1 18w - FDR p-value	G3 vs G1 EP - Log_2 presence ratio	G3 vs G1 EP - FDR p-value
[*Clostridium*] *innocuum* group	-0,26	0,9619	0,13	0,9743	0,35	0,9316
[*Desulfobacterium*] *catecholicum* group	-2,30	0,9619	-3,35	0,6756	-1,76	0,9316
[*Eubacterium*] *brachy* group	-0,71	0,9619	-1,52	0,5097	-0,94	0,9316
[*Eubacterium*] *coprostanoligenes* group	0,22	0,9619	0,59	0,9743	-0,29	0,9316
[*Eubacterium*] *eligens* group	-0,26	0,9619	0,14	0,9743	0,36	0,9316
[*Eubacterium*] *fissicatena* group	-0,26	0,9619	0,12	0,9743	0,34	0,9316
[*Eubacterium*] *hallii* group	1,29	0,9619	-1,37	0,9743	0,93	0,9316
[*Eubacterium*] *nodatum* group	0,95	0,9619	0,05	0,9743	2,35	0,9316
[*Eubacterium*] *oxidoreducens* group	-0,90	0,9619	-0,28	0,9743	-0,93	0,9316
[*Eubacterium*] *ruminantium* group	-0,03	0,9854	-0,47	0,9743	0,36	0,9316
[*Eubacterium*] *ventriosum* group	-4,38	0,4263	1,06	0,9743	-7,37	0,0015
[*Eubacterium*] *xylanophilum* group	-0,72	0,9619	-1,35	0,6486	-1,43	0,9316
[*Ruminococcus*] *gnavus* group	-0,26	0,9619	0,14	0,9743	-1,37	0,9316
[*Ruminococcus*] *torques* group	-1,82	0,9619	0,80	0,9743	0,68	0,9316
28-4	-0,26	0,9619	0,13	0,9743	3,68	0,9316
966-1	-0,26	0,9619	0,14	0,9743	0,36	0,9316
A2	0,97	0,9619	-1,02	0,9743	-0,40	0,9316
Acetatifactor	-0,74	0,9619	-2,14	0,3787	-0,48	0,9316
Acetitomaculum	-1,25	0,9619	-1,82	0,7164	-1,54	0,9316
Acetoanaerobium	-0,26	0,9619	-2,07	0,9514	0,36	0,9316
Acidaminobacter	-1,65	0,9619	0,14	0,9743	0,36	0,9316
Acidovorax	-0,26	0,9619	0,14	0,9743	0,36	0,9316
Acinetobacter	-1,73	0,9619	-4,56	0,3836	-0,64	0,9316
Actinomyces	-0,16	0,9619	0,42	0,9743	0,09	0,9687
Aerococcus	-0,26	0,9619	0,13	0,9743	0,35	0,9316
Aeromonas	-0,26	0,9619	1,38	0,9743	-0,75	0,9316
Agathobacter	2,00	0,9619	3,57	0,6796	1,53	0,9316
Akkermansia	-0,10	0,9619	0,21	0,9743	1,12	0,9316
Albidovulum	-0,26	0,9619	-1,35	0,9743	0,36	0,9316
Aliidiomarina	-0,26	0,9619	-2,07	0,9514	0,36	0,9316
Alistipes	0,43	0,9619	1,06	0,5906	0,37	0,9316
Alkalibacterium	-0,26	0,9619	-2,90	0,7164	-1,37	0,9316
Alkalicoccus	-1,60	0,9619	-3,06	0,7478	-1,75	0,9316
Alkaliflexus	-0,26	0,9619	0,14	0,9743	0,36	0,9316
Alkaliphilus	-0,26	0,9619	0,13	0,9743	0,36	0,9316
Allobacillus	-0,26	0,9619	0,14	0,9743	0,36	0,9316
Allobaculum	2,53	0,9619	-0,35	0,9743	-5,64	0,0314
Alloprevotella	0,25	0,9619	0,34	0,9743	0,90	0,9316
Allorhizobium-Neorhizobium-Pararhizobium-Rhizobium	-0,26	0,9619	0,14	0,9743	0,36	0,9316
Alloscardovia	-1,97	0,9619	-1,39	0,9514	-2,81	0,9316
Alpha proteobacterium GMD21G01	-0,26	0,9619	0,14	0,9743	0,36	0,9316
AlphaI cluster	-0,26	0,9619	0,14	0,9743	0,36	0,9316
Altererythrobacter	-0,26	0,9619	0,14	0,9743	0,36	0,9316
Anaerobacillus	-1,64	0,9619	-4,47	0,3945	0,35	0,9316
Anaerococcus	-0,26	0,9619	0,14	0,9743	1,56	0,9316
Anaerocolumna	-3,28	0,9619	-0,63	0,9743	-3,03	0,9316
Anaerofustis	-0,79	0,9619	-2,17	0,9514	-0,74	0,9316
Anaeroglobus	1,28	0,9619	0,14	0,9743	0,36	0,9316
Anaeroplasma	-0,87	0,9619	3,09	0,5906	-1,42	0,9316
Anaerosolibacter	-0,26	0,9619	0,14	0,9743	0,36	0,9316
Anaerosporobacter	-0,07	0,9768	-1,24	0,9743	-0,75	0,9316
Anaerostipes	0,80	0,9619	1,17	0,9743	0,28	0,9316
Anaerotruncus	-1,10	0,9619	-1,13	0,9506	0,07	0,9572
Anaerovorax	-0,26	0,9619	-1,27	0,5906	0,31	0,9316
Anditalea	-0,26	0,9619	-3,57	0,5655	-1,36	0,9316
Anoxynatronum	-0,26	0,9619	0,13	0,9743	0,36	0,9316
Arcanobacterium	-0,26	0,9619	3,10	0,7439	1,55	0,9316
Arenimonas	-0,26	0,9619	-1,40	0,9743	-1,76	0,9316
Arsenicitalea	-0,26	0,9619	0,14	0,9743	0,36	0,9316
Asaccharobacter	-1,70	0,9619	0,36	0,9743	-0,23	0,9316
ASF356	-0,05	0,9854	-2,45	0,7544	2,00	0,9316
Atopobium	-0,26	0,9619	0,13	0,9743	0,35	0,9316
Avena sativa (oat)	1,28	0,9619	0,14	0,9743	0,36	0,9316
Azoarcus	1,22	0,9619	-2,18	0,8792	-0,95	0,9316
Azospirillum sp. 47_25	0,31	0,9619	-2,34	0,5825	-0,33	0,9316
Bacillus	-0,26	0,9619	-1,33	0,9743	-1,44	0,9316
Bacteroidales bacterium 55_9	2,25	0,9619	0,14	0,9743	-2,87	0,9316
Bacteroides	-0,37	0,9619	0,32	0,9743	0,19	0,9316
Bifidobacterium	-0,99	0,9619	-0,48	0,9743	-0,43	0,9316

Bilophila	-1,15	0,9619	-1,70	0,7164	0,44	0,9316
Blastopirellula	-0,26	0,9619	0,13	0,9743	-1,36	0,9316
Blattella germanica (German cockroach)	-4,00	0,9619	1,23	0,9743	-4,68	0,9316
Blautia	-0,80	0,9619	-0,54	0,9743	-1,52	0,9316
Bradyrhizobium	-0,26	0,9619	0,14	0,9743	0,36	0,9316
Brevundimonas	0,70	0,9619	-3,06	0,7544	-0,84	0,9316
Butyricicoccus	-1,33	0,9619	-0,35	0,9743	-0,08	0,9517
Butyricimonas	-0,87	0,9619	1,08	0,6114	0,21	0,9316
Butyrivibrio	0,27	0,9619	1,24	0,9743	1,22	0,9316
Butyrivibrio 2	-0,26	0,9619	2,04	0,9743	0,36	0,9316
CAG-352	7,04	0,0078	3,63	0,3945	6,04	0,0309
Caldicoprobacter	-0,57	0,9619	2,57	0,3662	0,81	0,9316
Calothrix PCC-6303	-0,26	0,9619	-1,35	0,9743	2,20	0,9316
Campylobacter	-0,11	0,9678	0,14	0,9743	0,36	0,9316
Candidate division SR1 bacterium taxon 345	-0,26	0,9619	-1,35	0,9743	1,56	0,9316
Candidatus Alysiosphaera	-0,26	0,9619	0,14	0,9743	0,36	0,9316
Candidatus Amoebophilus	-0,26	0,9619	0,14	0,9743	0,36	0,9316
Candidatus Chlorothrix	-0,26	0,9619	0,14	0,9743	0,36	0,9316
Candidatus Endomicrobium	-0,26	0,9619	0,14	0,9743	0,36	0,9316
Candidatus Gastranaerophilales bacterium Zag_111	-0,11	0,9678	0,14	0,9743	0,36	0,9316
Candidatus Saccharimonas	1,05	0,9619	0,13	0,9743	-0,02	0,9861
Candidatus Soleaferrea	0,79	0,9619	-1,11	0,9743	0,66	0,9316
Candidatus Udaeobacter	-2,34	0,9619	0,14	0,9743	0,36	0,9316
Capnocytophaga	-0,26	0,9619	0,14	0,9743	0,48	0,9316
Cardiobacterium	-0,26	0,9619	0,14	0,9743	0,36	0,9316
Carya cathayensis	-0,26	0,9619	0,14	0,9743	0,36	0,9316
Catabacter	0,73	0,9619	1,33	0,9743	0,35	0,9316
Catenibacterium	-0,26	0,9619	1,44	0,9743	0,35	0,9316
Caulobacter	-0,26	0,9619	0,14	0,9743	0,36	0,9316
Cellulomonas	-0,26	0,9619	0,13	0,9743	0,36	0,9316
Cellulosimicrobium	-0,88	0,9619	-1,12	0,9743	-1,21	0,9316
Cesiribacter	-0,26	0,9619	0,14	0,9743	0,36	0,9316
Chara vulgaris	-0,26	0,9619	-2,07	0,9514	0,36	0,9316
Chiayiivirga	-0,26	0,9619	-2,07	0,9514	0,36	0,9316
Christensenellaceae R-7 group	-0,25	0,9619	1,22	0,7878	-0,04	0,9746
Chrysiogenes	-0,26	0,9619	0,14	0,9743	0,36	0,9316
Cloacibacterium	-0,26	0,9619	0,14	0,9743	-1,37	0,9316
Clostridium sensu stricto 1	-0,26	0,9619	0,13	0,9743	2,18	0,9316
Collinsella	-1,65	0,9619	1,38	0,9743	-0,75	0,9316
Constrictibacter	-0,26	0,9619	0,14	0,9743	0,36	0,9316
Coprococcus 2	-2,55	0,8287	-3,25	0,2784	-2,66	0,8720
Coprococcus 3	-0,87	0,9619	-0,29	0,9743	-0,32	0,9316
Coriobacteriaceae UCG-002	0,67	0,9619	-1,38	0,9121	1,44	0,9316
Corynebacterium	0,62	0,9619	-2,07	0,9514	2,20	0,9316
Corynebacterium 1	-1,65	0,9619	0,14	0,9743	0,36	0,9316
Cryomorpha	-0,26	0,9619	0,14	0,9743	0,36	0,9316
Cutibacterium	-0,71	0,9619	-1,32	0,9514	-1,31	0,9316
Defluviitaleaceae UCG-011	1,58	0,9619	1,64	0,6796	1,44	0,9316
Delftia	-0,26	0,9619	0,14	0,9743	0,36	0,9316
Desulfatitalea	-0,26	0,9619	0,14	0,9743	-0,75	0,9316
Desulfovibrio	-0,72	0,9619	-0,97	0,9514	0,16	0,9316
Devosia	-0,26	0,9619	0,14	0,9743	0,36	0,9316
Dialister	-0,26	0,9619	-3,22	0,3945	-0,72	0,9316
Dietzia	2,85	0,9619	-2,07	0,9514	0,36	0,9316
Diplosphaera	-0,26	0,9619	-1,35	0,9743	0,36	0,9316
DNF00809	-0,75	0,9619	-1,80	0,4575	-0,66	0,9316
Dorea	4,01	0,6721	-3,39	0,4291	0,09	0,9687
Dubosiella	-0,39	0,9619	0,60	0,9743	-2,75	0,6586
Egicoccus	-0,16	0,9628	-3,63	0,5906	-2,05	0,9316
Eisenbergiella	0,93	0,9619	0,33	0,9743	-0,82	0,9316
Elusimicrobium	0,35	0,9619	4,32	0,2784	-1,02	0,9316
Enhygromyxa sp. SIS-1	-0,26	0,9619	0,14	0,9743	0,36	0,9316
Ensifer	-2,34	0,9619	0,14	0,9743	0,36	0,9316
Enterococcus	2,22	0,9619	1,23	0,9514	-1,41	0,9316
Enterorhabdus	-0,07	0,9619	-0,47	0,9743	0,66	0,9316
Erysipelatoclostridium	-0,26	0,9624	0,46	0,9743	1,58	0,9316
Erysipelothrix	-0,26	0,9619	-2,54	0,8358	0,36	0,9316
Erythrobacter	-1,65	0,9619	-1,35	0,9743	0,36	0,9316
Escherichia-Shigella	-3,83	0,6721	1,67	0,9514	-3,07	0,9316
Eubacterium	-0,26	0,9619	3,12	0,8448	1,56	0,9316

Euglena deses	-0,26	0,9619	-2,09	0,9514	0,36	0,9316
Euzebya	-0,26	0,9619	0,14	0,9743	0,36	0,9316
F0058	-0,26	0,9619	-0,11	0,9743	0,36	0,9316
Facklamia	-0,26	0,9619	0,14	0,9743	0,36	0,9316
Faecalibacterium	-0,26	0,9619	0,14	0,9743	0,36	0,9316
Faecalibaculum	-0,67	0,9619	1,19	0,9514	-1,03	0,9316
Family XIII AD3011 group	-0,44	0,9619	-0,44	0,9743	-0,12	0,9316
Family XIII UCG-001	-1,05	0,9619	-1,77	0,4858	-0,03	0,9786
Filifactor	-0,26	0,9619	-2,90	0,7065	0,36	0,9316
Fistulifera solaris	-0,26	0,9619	0,14	0,9743	0,36	0,9316
Flavitalea	-0,26	0,9619	0,14	0,9743	0,36	0,9316
Flavobacterium	-0,26	0,9619	-1,28	0,9743	-0,75	0,9316
Flexilinea	1,28	0,9619	0,14	0,9743	0,36	0,9316
Fluviicola	1,15	0,9619	-4,28	0,3945	0,35	0,9316
Fluviimonas	-0,26	0,9619	0,14	0,9743	0,36	0,9316
Fournierella	-0,26	0,9619	0,14	0,9743	0,36	0,9316
Fretibacterium	-0,26	0,9619	-2,07	0,9514	-0,75	0,9316
Fusibacter	-0,26	0,9619	0,14	0,9743	0,36	0,9316
Fusobacterium	-0,35	0,9619	-2,49	0,3945	0,36	0,9316
GCA-900066225	0,82	0,9619	-0,55	0,9743	0,49	0,9316
GCA-900066575	-0,63	0,9619	0,35	0,9743	0,07	0,9446
Gemella	-0,26	0,9619	0,14	0,9743	0,36	0,9316
Gemmatimonas	-0,26	0,9619	0,14	0,9743	0,36	0,9316
Gemmobacter	-0,26	0,9619	-2,54	0,8358	0,36	0,9316
Geobacter	-0,26	0,9619	-2,07	0,9514	0,36	0,9316
Gerbera hybrid cultivar	1,28	0,9619	0,14	0,9743	0,36	0,9316
Gillisia	1,20	0,9619	-4,51	0,4291	-0,65	0,9316
Gut metagenome-01	-0,06	0,9678	0,14	0,9743	0,36	0,9316
Gut metagenome-02	-0,90	0,9619	-0,04	0,9814	-1,59	0,9316
Gut metagenome-03	0,71	0,9619	2,01	0,3945	0,64	0,9316
Gut metagenome-04	2,01	0,9619	0,14	0,9743	-1,37	0,9316
Haliea	-0,26	0,9619	0,14	0,9743	0,36	0,9316
Halomonas	-3,82	0,9619	-5,96	0,2784	-2,66	0,9316
Haloplasma	-0,26	0,9619	0,14	0,9743	0,36	0,9316
Harryflintia	-0,42	0,9619	-0,94	0,9514	0,22	0,9316
Helicobacter	0,58	0,9619	1,60	0,4575	1,45	0,9316
Hoeflea	-0,26	0,9619	-2,12	0,9743	0,35	0,9316
Howardella	-0,26	0,9619	0,13	0,9743	0,36	0,9316
Hungatella	-0,26	0,9619	0,14	0,9743	0,36	0,9316
Hydrogenoanaerobacterium	-0,26	0,9619	-2,54	0,8358	0,45	0,9316
Hydrogenophaga	1,28	0,9619	-1,30	0,9743	-1,34	0,9316
hydrothermal vent metagenome	-0,26	0,9619	0,14	0,9743	0,36	0,9316
Hyphomonas	-0,26	0,9619	0,14	0,9743	0,36	0,9316
Hypnocyclicus	-0,26	0,9619	-2,07	0,9514	0,36	0,9316
Iamia	-0,26	0,9619	0,14	0,9743	0,36	0,9316
Ileibacterium	-0,26	0,9619	0,14	0,9743	1,56	0,9316
Ilumatobacter	-0,26	0,9619	-4,07	0,4717	-2,10	0,9316
IMCC26134	-0,26	0,9619	0,14	0,9743	0,36	0,9316
IMCC26207	-0,26	0,9619	0,14	0,9743	0,36	0,9316
Incertae Sedis	-0,26	0,9619	0,14	0,9743	0,36	0,9316
Inquilinus	-0,26	0,9619	0,14	0,9743	0,36	0,9316
Intestinimonas	-0,30	0,9619	-0,37	0,9743	-0,39	0,9316
Jeotgalicoccus	-0,26	0,9619	0,14	0,9743	0,36	0,9316
Johnsonella	2,01	0,9619	0,14	0,9743	0,36	0,9316
JTB255 marine benthic group	-0,26	0,9619	-1,38	0,9743	0,35	0,9316
Kryptoperidinium foliaceum	-0,26	0,9619	-1,37	0,9743	0,36	0,9316
Lachnoclostridium	-2,20	0,6149	-0,11	0,9743	-0,19	0,9316
Lachnoclostridium 10	-0,26	0,9619	0,14	0,9743	-1,37	0,9316
Lachnoclostridium 12	-0,26	0,9619	0,14	0,9743	-1,37	0,9316
Lachnoclostridium 5	-0,26	0,9619	0,14	0,9743	-1,37	0,9316
Lachnospiraceae AC2044 group	-1,65	0,9619	-0,16	0,9743	2,20	0,9316
Lachnospiraceae FCS020 group	0,14	0,9619	-0,02	0,9806	0,33	0,9316
Lachnospiraceae NC2004 group	1,31	0,9619	-1,53	0,9040	-2,10	0,9316
Lachnospiraceae NK4A136 group	-0,97	0,9619	-1,07	0,5825	-0,19	0,9316
Lachnospiraceae NK4B4 group	2,28	0,9619	-3,83	0,3662	-4,06	0,6586
Lachnospiraceae UCG-001	-2,20	0,9512	-0,81	0,9743	-1,16	0,9316
Lachnospiraceae UCG-002	0,32	0,9619	-2,42	0,3662	-2,83	0,3801
Lachnospiraceae UCG-004	-4,15	0,9512	1,44	0,9743	-8,10	0,0059
Lachnospiraceae UCG-006	-1,75	0,6721	0,46	0,9743	-0,33	0,9316
Lachnospiraceae UCG-008	-0,64	0,9619	-0,25	0,9743	0,69	0,9316

Lachnospiraceae UCG-009	-1,67	0,9619	-0,05	0,9806	-0,06	0,9746
Lachnospiraceae UCG-010	0,80	0,9619	-2,69	0,4575	0,45	0,9316
Lachnotalea	-0,26	0,9619	0,14	0,9743	0,36	0,9316
Lactobacillus	-0,94	0,9619	1,16	0,9514	1,92	0,9316
Lawsonella	-2,34	0,9619	0,14	0,9743	1,09	0,9316
Legionella	-0,26	0,9619	0,14	0,9743	0,36	0,9316
Lentimicrobium	-0,26	0,9619	0,14	0,9743	0,36	0,9316
Leptothrix	-0,26	0,9619	0,14	0,9743	0,36	0,9316
Leptotrichia	-0,26	0,9619	-1,35	0,9743	0,36	0,9316
Leucobacter	-0,26	0,9619	-2,07	0,9514	0,36	0,9316
Lewinella	-0,26	0,9619	0,14	0,9743	0,36	0,9316
Loktanella	-0,26	0,9619	-3,85	0,5228	0,35	0,9316
Luteimonas	-0,26	0,9619	0,13	0,9743	0,35	0,9316
Luteococcus	-0,26	0,9619	0,14	0,9743	0,36	0,9316
Luteolibacter	-3,19	0,9619	1,28	0,9743	-1,72	0,9316
Lutispora	-0,26	0,9619	0,14	0,9743	0,36	0,9316
Marmoricola	-2,34	0,9619	0,14	0,9743	0,36	0,9316
Marvinbryantia	-0,18	0,9619	-1,25	0,9514	0,22	0,9316
Massilia	-0,26	0,9619	0,14	0,9743	-1,37	0,9316
Megasphaera	0,18	0,9619	0,15	0,9743	-1,11	0,9316
Mesorhizobium	-0,26	0,9619	0,14	0,9743	0,36	0,9316
Metagenome-01	-0,26	0,9619	0,14	0,9743	0,36	0,9316
Metagenome-02	-0,26	0,9619	-2,07	0,9514	0,36	0,9316
Metagenome-03	-0,26	0,9619	0,14	0,9743	0,36	0,9316
Metagenome-04	-0,26	0,9619	0,14	0,9743	0,36	0,9316
Metagenome-05	1,28	0,9619	-1,35	0,9743	0,36	0,9316
Methanosarcina	-0,26	0,9619	0,14	0,9743	0,36	0,9316
Methanosphaera	-0,26	0,9619	0,13	0,9743	-1,82	0,9316
Methylobacterium	-0,26	0,9619	0,14	0,9743	0,36	0,9316
Methylocystis	-0,26	0,9619	-2,07	0,9514	0,36	0,9316
Methylophilus	-0,26	0,9619	0,14	0,9743	0,36	0,9316
Mitsuaria	-0,26	0,9619	0,14	0,9743	0,36	0,9316
Mobilitalea	-0,26	0,9619	-1,35	0,9743	0,36	0,9316
Mogibacterium	-2,34	0,9619	0,14	0,9743	0,36	0,9316
Mongoliibacter	-0,26	0,9619	0,14	0,9743	0,36	0,9316
Mongoliitalea	-0,26	0,9619	0,14	0,9743	0,36	0,9316
Morganella	-2,39	0,9619	1,40	0,9743	-0,97	0,9316
Moryella	0,07	0,9678	0,97	0,9743	2,63	0,9316
Mouse gut metagenome	-0,26	0,9619	0,14	0,9743	0,36	0,9316
Mucispirillum	0,58	0,9619	2,03	0,3945	0,29	0,9316
Murdochiella	-0,26	0,9619	0,55	0,9743	0,45	0,9316
Muribaculum	-1,29	0,9619	0,28	0,9743	-0,10	0,9316
Mycoplasma	-0,26	0,9619	-2,07	0,9514	0,36	0,9316
Natronohydrobacter	-0,02	0,9938	-5,99	0,2784	-0,60	0,9316
Negativibacillus	-1,27	0,9619	1,38	0,9743	-0,75	0,9316
Nesterenkonia	-0,26	0,9619	-4,96	0,3787	-0,74	0,9316
Nicotiana benthamiana	-1,65	0,9619	0,14	0,9743	0,36	0,9316
Nitriliruptor	-2,34	0,9619	0,14	0,9743	0,36	0,9316
Nitrincola	-0,26	0,9619	0,14	0,9743	-0,75	0,9316
Nitrobacter	-0,26	0,9619	0,14	0,9743	0,36	0,9316
Nitrosomonas	-0,26	0,9619	0,14	0,9743	0,36	0,9316
Nocardioides	-0,26	0,9619	0,14	0,9743	0,36	0,9316
Nodosilinea PCC-7104	0,94	0,9619	-4,92	0,3945	-2,20	0,9316
Nodularia PCC-9350	-0,26	0,9619	0,14	0,9743	0,36	0,9316
Oceanobacillus	-0,26	0,9619	0,14	0,9743	0,36	0,9316
Ochrobactrum	-0,26	0,9619	-2,07	0,9514	0,36	0,9316
Odoribacter	1,50	0,9619	-2,82	0,4631	2,19	0,9316
Olsenella	-0,07	0,9678	-0,82	0,9743	-1,37	0,9316
Oryzihumus	-0,26	0,9619	0,14	0,9743	-1,37	0,9316
Oscillatoria PCC-6304	-0,26	0,9619	-0,35	0,9743	-1,31	0,9316
Oscillibacter	-0,95	0,9619	-0,22	0,9743	-0,37	0,9316
Oscillospira	0,06	0,9678	-1,02	0,9514	-0,21	0,9316
Oxalobacter	-0,37	0,9619	0,66	0,9514	0,02	0,9780
Paludibacter	-0,26	0,9619	0,14	0,9743	0,36	0,9316
Paludibaculum	2,01	0,9619	0,14	0,9743	0,36	0,9316
Papillibacter	1,04	0,9619	2,74	0,6796	2,84	0,9316
Parabacteroides	-0,61	0,9619	0,76	0,9514	-0,26	0,9316
Paracoccus	-0,26	0,9619	0,14	0,9743	0,36	0,9316
Paradevosia	-0,26	0,9619	0,14	0,9743	0,36	0,9316
Parasutterella	-0,04	0,9805	-2,85	0,3945	-0,27	0,9316

Parvibacter	0,58	0,9619	-2,29	0,3945	-0,93	0,9316
Parvimonas	-0,26	0,9619	-2,07	0,9514	1,56	0,9316
Pelomonas	-0,26	0,9619	-2,90	0,7065	0,36	0,9316
Peptococcus	-0,13	0,9619	-1,89	0,3012	-0,81	0,9316
Peptoniphilus	-0,26	0,9619	0,14	0,9743	0,36	0,9316
Peptostreptococcus	-0,26	0,9619	0,13	0,9743	0,35	0,9316
Peredibacter	-0,26	0,9619	0,14	0,9743	0,36	0,9316
Phaeobacter	-0,26	0,9619	0,14	0,9743	0,36	0,9316
Phascolarctobacterium	-0,26	0,9619	0,14	0,9743	0,36	0,9316
Phocaeicola	-0,26	0,9619	2,04	0,9743	-1,37	0,9316
Phyllobacterium	-0,26	0,9619	0,14	0,9743	-1,37	0,9316
Pinus sylvestris (Scots pine)	1,25	0,9619	2,07	0,9743	-0,75	0,9316
Pirellula	-0,26	0,9619	0,14	0,9743	0,36	0,9316
Planktosalinus	1,25	0,9619	-0,11	0,9743	-1,36	0,9316
Planococcus	-0,26	0,9619	-4,49	0,3945	0,35	0,9316
Planoglabratella opercularis	-0,26	0,9619	0,13	0,9743	0,36	0,9316
Planomicrobium	-0,26	0,9619	-4,94	0,3593	0,35	0,9316
Plesiomonas	-0,26	0,9619	0,14	0,9743	0,36	0,9316
Polaromonas	-0,26	0,9619	0,13	0,9743	0,35	0,9316
Pontibacter	-0,26	0,9619	-1,35	0,9743	-0,75	0,9316
Porphyrobacter	-1,64	0,9619	-3,60	0,5825	-0,75	0,9316
Porphyromonas	-0,81	0,9619	-5,62	0,2784	-0,68	0,9316
Possible genus Sk018	1,28	0,9619	0,14	0,9743	2,64	0,9316
Prevotella	1,30	0,9619	-1,42	0,9514	2,63	0,9316
Prevotella 2	-0,26	0,9619	0,14	0,9743	0,36	0,9316
Prevotella 7	-0,26	0,9619	-1,36	0,9743	-0,17	0,9417
Prevotellaceae UCG-001	-0,45	0,9619	0,92	0,9506	0,24	0,9316
Prevotellaceae UCG-003	-0,47	0,9619	1,02	0,9514	0,45	0,9316
Prosthecobacter	-0,26	0,9619	-1,35	0,9743	0,36	0,9316
Proteiniclasticum	-0,26	0,9619	-1,35	0,9743	0,36	0,9316
Proteus	-2,63	0,9619	0,31	0,9743	0,35	0,9316
Pseudohongiella	-0,26	0,9619	0,14	0,9743	-0,75	0,9316
Pseudolabrys	-0,26	0,9619	0,14	0,9743	0,36	0,9316
Pseudomonas	-0,26	0,9619	-3,56	0,5655	0,35	0,9316
Pseudoxanthomonas	-0,26	0,9619	0,14	0,9743	0,36	0,9316
Pygmaiobacter	-0,62	0,9619	0,26	0,9743	1,55	0,9316
Pyramidobacter	-0,26	0,9619	0,14	0,9743	-0,75	0,9316
Rheinheimera	-0,26	0,9619	0,14	0,9743	0,36	0,9316
Rhodobaca	-0,26	0,9619	0,14	0,9743	0,36	0,9316
Rhodobaculum	-0,26	0,9619	-2,07	0,9514	0,36	0,9316
Rhodonellum	1,28	0,9619	-1,35	0,9743	-0,75	0,9316
Rhodopirellula	-1,59	0,9619	0,13	0,9743	0,35	0,9316
Rikenella	1,47	0,9619	-3,21	0,2784	0,09	0,9573
Rikenellaceae RC9 gut group	0,33	0,9619	0,12	0,9743	0,52	0,9316
Robinsoniella	-0,26	0,9619	0,14	0,9743	0,36	0,9316
Roseburia	-0,72	0,9619	-0,40	0,9743	-0,62	0,9316
Roseiarcus	-0,26	0,9619	0,14	0,9743	0,36	0,9316
Roseicyclus	-0,26	0,9619	0,14	0,9743	0,36	0,9316
Roseococcus	1,28	0,9619	0,14	0,9743	0,36	0,9316
Rothia	-0,26	0,9619	0,14	0,9743	-1,37	0,9316
Rubribacterium	-0,26	0,9619	0,14	0,9743	0,36	0,9316
Rubrobacter	-0,26	0,9619	0,14	0,9743	0,36	0,9316
Ruminiclostridium	-0,47	0,9619	-0,63	0,9743	0,01	0,9889
Ruminiclostridium 1	-0,34	0,9619	1,29	0,9121	0,72	0,9316
Ruminiclostridium 5	-0,90	0,9619	-0,82	0,9514	0,24	0,9316
Ruminiclostridium 6	-0,35	0,9619	-0,92	0,8358	0,18	0,9316
Ruminiclostridium 9	-0,70	0,9619	-0,71	0,9514	0,52	0,9316
Ruminococcaceae NK4A214 group	-1,12	0,9619	-0,91	0,8358	-0,72	0,9316
Ruminococcaceae UCG-002	-0,26	0,9619	-0,46	0,9743	1,53	0,9316
Ruminococcaceae UCG-003	-1,72	0,6721	-0,27	0,9743	-0,32	0,9316
Ruminococcaceae UCG-004	-3,64	0,6149	-2,35	0,5004	-1,28	0,9316
Ruminococcaceae UCG-005	-0,07	0,9619	0,31	0,9743	-0,77	0,9316
Ruminococcaceae UCG-007	-1,65	0,9619	1,38	0,9743	1,56	0,9316
Ruminococcaceae UCG-009	-0,31	0,9619	-1,10	0,6756	-0,99	0,9316
Ruminococcaceae UCG-010	-0,22	0,9619	1,66	0,4259	0,77	0,9316
Ruminococcaceae UCG-013	0,28	0,9619	0,62	0,9743	0,10	0,9417
Ruminococcaceae UCG-014	0,30	0,9619	0,41	0,9743	0,37	0,9316
Ruminococcus 1	-0,51	0,9619	0,38	0,9743	-0,24	0,9316
Ruminococcus 2	-8,97	0,0000	-4,75	0,2784	-0,14	0,9517
S5-A14a	-0,26	0,9619	0,14	0,9743	0,36	0,9316

Shuttleworthia	-0,26	0,9619	0,14	0,9743	0,36	0,9316
Solibacillus	-0,26	0,9619	0,14	0,9743	0,36	0,9316
Sphingomonas	-0,12	0,9678	0,31	0,9743	0,36	0,9316
Sphingopyxis	-0,26	0,9619	0,14	0,9743	0,36	0,9316
Sphingosinicella	-0,26	0,9619	0,14	0,9743	0,36	0,9316
Spirochaeta 2	-0,26	0,9619	0,14	0,9743	0,36	0,9316
Spirogyra pratensis	-0,26	0,9619	-2,19	0,9533	0,35	0,9316
Spirogyra sp. CLP-2000	-0,26	0,9619	0,14	0,9743	0,36	0,9316
Sporosarcina	2,47	0,9619	0,12	0,9743	0,35	0,9316
Staphylococcus	0,60	0,9619	0,45	0,9743	0,09	0,9662
Streptococcus	-1,17	0,9619	0,86	0,9743	-1,95	0,9316
Streptomyces	-1,62	0,9619	-1,38	0,9743	0,35	0,9316
Subdoligranulum	-3,57	0,9619	-1,66	0,9514	-4,14	0,9316
Subsaxibacter	-0,26	0,9619	0,14	0,9743	0,36	0,9316
Sulfurimonas	-0,26	0,9619	0,14	0,9743	0,36	0,9316
Sutterella	-1,63	0,9619	2,30	0,9743	-0,42	0,9316
Tabrizicola	-0,26	0,9619	-2,07	0,9514	0,36	0,9316
Tannerella	-0,26	0,9619	-4,00	0,4575	-2,61	0,9316
Thalassobius	-0,26	0,9619	-2,07	0,9514	0,36	0,9316
Thioalkalivibrio	1,28	0,9619	-3,25	0,6756	0,35	0,9316
Thiocapsa	-0,26	0,9619	-4,19	0,4575	0,35	0,9316
Thioflexothrix	-0,26	0,9619	-1,38	0,9743	-1,37	0,9316
Tissierella	-0,26	0,9619	-1,38	0,9743	0,35	0,9316
Treponema 2	-1,27	0,9619	-1,30	0,9514	0,48	0,9316
Tropicimonas	-0,26	0,9619	-2,92	0,7439	0,36	0,9316
Truepera	-0,26	0,9619	-2,92	0,7439	-0,75	0,9316
Turicibacter	-0,39	0,9619	-3,59	0,2784	-1,67	0,9316
Tyzzerella	-1,00	0,9619	-1,36	0,8358	-0,31	0,9316
Tyzzerella 3	2,75	0,9619	0,71	0,9743	0,42	0,9316
Tyzzerella 4	-1,65	0,9619	0,14	0,9743	0,36	0,9316
UBA1819	-1,43	0,9619	0,46	0,9743	-0,36	0,9316
UC5-1-2E3	-0,26	0,9619	0,14	0,9743	0,36	0,9316
Uncultured *Acidobacteriaceae* bacterium	-2,34	0,9619	0,14	0,9743	0,36	0,9316
Uncultured *Actinomycetaceae* bacterium	-0,26	0,9619	0,14	0,9743	0,36	0,9316
Uncultured *Aquiflexum* sp.	-1,65	0,9619	-2,11	0,9514	0,35	0,9316
Uncultured bacterium-01	-0,26	0,9619	0,14	0,9743	0,36	0,9316
Uncultured bacterium-02	-1,06	0,9619	-1,77	0,7439	1,98	0,9316
Uncultured bacterium-03	-0,26	0,9619	0,14	0,9743	0,36	0,9316
Uncultured bacterium-04	-0,26	0,9619	0,14	0,9743	-1,80	0,9316
Uncultured bacterium-05	2,73	0,9619	-5,45	0,3548	-1,47	0,9316
Uncultured bacterium-06	-0,26	0,9619	0,14	0,9743	-1,80	0,9316
Uncultured bacterium-07	0,04	0,9619	0,15	0,9743	0,43	0,9316
Uncultured bacterium-08	-0,26	0,9619	-3,53	0,5228	0,35	0,9316
Uncultured bacterium-09	-0,26	0,9619	-1,35	0,9743	0,36	0,9316
Uncultured bacterium-10	-0,26	0,9619	-1,35	0,9743	0,36	0,9316
Uncultured bacterium-11	1,22	0,9619	-3,37	0,6796	-0,74	0,9316
Uncultured bacterium-12	-0,26	0,9619	-2,07	0,9514	0,36	0,9316
Uncultured bacterium-13	-0,26	0,9619	0,14	0,9743	0,36	0,9316
Uncultured bacterium-14	-0,26	0,9619	0,14	0,9743	0,36	0,9316
Uncultured bacterium-15	-0,26	0,9619	0,14	0,9743	0,36	0,9316
Uncultured bacterium-16	-0,26	0,9619	0,14	0,9743	0,36	0,9316
Uncultured bacterium-17	-0,11	0,9678	-0,69	0,9743	0,35	0,9316
Uncultured bacterium-18	-0,26	0,9619	0,14	0,9743	0,36	0,9316
Uncultured bacterium-19	-0,34	0,9619	0,44	0,9743	0,24	0,9316
Uncultured bacterium-20	2,01	0,9619	1,38	0,9743	-0,75	0,9316
Uncultured bacterium-21	0,05	0,9678	-0,18	0,9743	1,15	0,9316
Uncultured bacterium-22	0,82	0,9619	-2,40	0,4858	0,56	0,9316
Uncultured bacterium-23	2,01	0,9619	0,14	0,9743	0,36	0,9316
Uncultured bacterium-24	-0,26	0,9619	-1,35	0,9743	0,36	0,9316
Uncultured bacterium-25	-0,26	0,9619	0,14	0,9743	0,36	0,9316
Uncultured bacterium-26	-0,26	0,9619	-1,35	0,9743	-0,75	0,9316

Uncultured bacterium-27	-0,26	0,9619	-2,07	0,9514	0,36	0,9316
Uncultured bacterium-28	-0,26	0,9619	-1,35	0,9743	-0,75	0,9316
Uncultured bacterium-29	-0,26	0,9619	-0,13	0,9743	-1,80	0,9316
Uncultured bacterium-30	-0,26	0,9619	0,14	0,9743	0,36	0,9316
Uncultured bacterium-31	-0,26	0,9619	0,14	0,9743	0,36	0,9316
Uncultured bacterium-32	1,28	0,9619	0,14	0,9743	1,56	0,9316
Uncultured bacterium-33	0,56	0,9619	1,41	0,9506	-0,68	0,9316
Uncultured bacterium-34	-0,26	0,9619	0,14	0,9743	0,36	0,9316
Uncultured bacterium-35	1,28	0,9619	-2,92	0,7164	0,36	0,9316
Uncultured bacterium-36	-0,26	0,9619	0,14	0,9743	0,36	0,9316
Uncultured bacterium-37	-0,26	0,9619	0,14	0,9743	0,36	0,9316
Uncultured bacterium-38	0,76	0,9619	0,77	0,9743	0,74	0,9316
Uncultured bacterium-39	-0,26	0,9619	0,14	0,9743	0,36	0,9316
Uncultured *Bacteroidales* bacterium	-0,41	0,9619	0,44	0,9743	-0,07	0,9497
Uncultured *Bacteroidetes* bacterium	-0,26	0,9619	-3,64	0,4858	-2,40	0,9316
Uncultured candidate division SR1 bacterium	-0,26	0,9619	0,14	0,9743	0,36	0,9316
Uncultured *Chloroflexi* bacterium	-0,26	0,9619	0,14	0,9743	0,36	0,9316
Uncultured *Clostridiales* bacterium	-1,54	0,9619	-0,48	0,9743	-0,33	0,9316
Uncultured cyanobacterium	-0,26	0,9619	0,14	0,9743	0,36	0,9316
Uncultured *Erysipelotrichaceae* bacterium	-8,02	0,1525	8,47	0,1811	-1,35	0,9316
Uncultured eukaryote	1,21	0,9619	-3,47	0,6796	0,10	0,9671
Uncultured gamma proteobacterium	-0,26	0,9619	-2,54	0,8439	0,36	0,9316
Uncultured *Lachnospiraceae* bacterium	1,45	0,9619	3,78	0,2784	2,07	0,9316
Uncultured *Microgenomates* group bacterium	-0,26	0,9619	0,14	0,9743	0,36	0,9316
Uncultured organism	0,75	0,9619	-3,76	0,2784	-0,16	0,9387
Uncultured phototrophic eukaryote	5,02	0,9619	0,11	0,9755	4,17	0,9316
Uncultured prokaryote	-0,26	0,9619	0,14	0,9743	0,36	0,9316
Uncultured rumen bacterium-01	1,57	0,9619	2,21	0,7164	-2,79	0,9316
Uncultured rumen bacterium-02	-0,26	0,9619	1,38	0,9743	1,56	0,9316
Uncultured rumen bacterium-03	-0,31	0,9619	-0,76	0,9743	-0,14	0,9316
Uncultured rumen bacterium-04	1,32	0,9619	-2,38	0,4858	0,35	0,9316
Uncultured soil bacterium	-0,26	0,9619	0,14	0,9743	-1,37	0,9316
Uncultured *Sphingobacteriia* bacterium	-0,26	0,9619	0,14	0,9743	0,36	0,9316
Uncultured *Verrucomicrobia* bacterium	-2,34	0,9619	0,14	0,9743	0,36	0,9316
Uncultured-01	-0,26	0,9619	0,14	0,9743	0,36	0,9316
Uncultured-02	1,28	0,9619	-2,06	0,9514	-0,17	0,9417
Uncultured-03	-0,39	0,9619	0,34	0,9743	1,56	0,9316
Uncultured-04	-0,26	0,9619	0,14	0,9743	0,36	0,9316
Uncultured-05	-0,26	0,9619	-2,07	0,9514	0,36	0,9316
Uncultured-06	-0,26	0,9619	0,14	0,9743	0,36	0,9316
Uncultured-07	-0,26	0,9619	-2,07	0,9514	0,36	0,9316
Uncultured-08	-0,26	0,9619	0,13	0,9743	-0,53	0,9316
Uncultured-09	-0,26	0,9619	-3,04	0,7439	-0,75	0,9316
Uncultured-10	-1,12	0,9619	1,00	0,9514	0,35	0,9316
Uncultured-11	-0,26	0,9619	0,13	0,9743	0,35	0,9316
Uncultured-12	0,45	0,9619	0,68	0,9743	0,48	0,9316
Uncultured-13	0,51	0,9619	1,59	0,2784	1,08	0,9316
Uncultured-14	-1,05	0,9619	-1,70	0,3945	-1,21	0,9316
Uncultured-15	-1,18	0,9619	-0,94	0,8448	0,51	0,9316
Uncultured-16	0,84	0,9619	0,69	0,9743	0,24	0,9316
Uncultured-17	-0,26	0,9619	0,14	0,9743	0,36	0,9316
Uncultured-18	-0,26	0,9619	0,14	0,9743	1,56	0,9316
Uncultured-19	-0,26	0,9619	0,14	0,9743	0,36	0,9316
Uncultured-20	-0,26	0,9619	0,14	0,9743	0,36	0,9316
Uncultured-21	-0,26	0,9619	-2,54	0,8358	0,36	0,9316
Uncultured-22	-0,26	0,9619	0,14	0,9743	0,36	0,9316
Uncultured-23	1,02	0,9619	-3,99	0,5186	-1,74	0,9316
Uncultured-24	-0,26	0,9619	-2,07	0,9514	0,36	0,9316
Uncultured-25	-0,26	0,9619	-2,07	0,9514	0,36	0,9316
Uncultured-26	-0,80	0,9619	-1,02	0,7544	-0,15	0,9316
Uncultured-27	-0,26	0,9619	0,14	0,9743	-0,75	0,9316
Uncultured-28	-0,26	0,9619	0,14	0,9743	0,36	0,9316
Uncultured-29	-0,26	0,9619	0,14	0,9743	0,36	0,9316
Uncultured-30	-0,72	0,9619	-3,52	0,4245	-1,83	0,9316
Uncultured-31	-0,26	0,9619	0,14	0,9743	0,36	0,9316

Uncultured-32	-0,26	0,9619	-1,35	0,9743	-1,37	0,9316
Unidentified	0,69	0,9619	0,85	0,9743	0,86	0,9316
Veillonella	-0,26	0,9619	0,14	0,9743	-1,37	0,9316
Victivallis	-0,26	0,9619	0,14	0,9743	0,36	0,9316
Vitis vinifera (wine grape)	-0,26	0,9619	0,14	0,9743	0,45	0,9316
W5053	-0,26	0,9619	-2,92	0,7164	0,36	0,9316
Wolbachia	-0,26	0,9619	0,13	0,9743	0,36	0,9316
